# Tumor Mechanical Remodeling via Bidirectional Calcium Redistribution Enables Deep Photothermal‐Immunotherapy

**DOI:** 10.1002/advs.77625

**Published:** 2026-09-08

**Authors:** Hongjuan Zhao, Jinpei Sun, Chenxi Zhao, Yuting Cang, Keyu Zong, Yuxin Guo, Yajing Wang, Di Meng, Qingling Song, Lei Wang

**Affiliations:** ^1^ School of Pharmaceutical Sciences Zhengzhou University Zhengzhou China; ^2^ Key Laboratory of Nanomedicine for Targeting Diagnosis and Treatment Ministry of Education of China Zhengzhou University Zhengzhou China; ^3^ School of Pharmaceutical Sciences State Key Laboratory of Antiviral Drugs Pingyuan Laboratory Zhengzhou University Zhengzhou China

**Keywords:** calcium, deep penetration, mechanical remodeling, oxidative stress, photothermal‐immunotherapy

## Abstract

Ca^2+^ signaling serves as a core regulator controlling both cellular survival and immunomodulation pathways. Here, we report a paradigm‐shifting bidirectional Ca^2+^ redistribution nanomodulator (RP@AA‐Lip) that rewires Ca^2+^ homeostasis across intracellular‐extracellular compartments to orchestrate a self‐reinforcing mechanical‐immunological cascade for deep photothermal‐immunotherapy (PTI). This core‐shell nanomodulator RP@AA‐Lip consists of hypoxia‐targeting Rhodopseudomonas palustris (RP) as the photothermal core and an ascorbic acid (AA)‐enriched liposome (Lip) shell. Under NIR irradiation, RP activates TRPV1‐mediated Ca^2+^ influx, while AA inhibits PMCA4‐driven Ca^2+^ efflux, jointly triggering intracellular Ca^2+^ overload to induce mitochondrial damage and thermoresistance reversal for amplified immunogenic cell death. We find that extracellular Ca^2+^ depletion disassembles E‐cadherin junctions and softens tumor stiffness 4.23‐fold, enabling deep tumor penetration (up to 500 µm) and robust cytotoxic T cell (CTL) infiltration. Concurrently, the intracellular Ca^2+^ overload further stiffens tumor cells to establish mechanical immunosurveillance that improves CTL‐mediated tumor killing. In triple‐negative breast cancer models, this integrated strategy elicits a robust antitumor immune cascade to remodel the tumor microenvironment and suppress tumor progression. Collectively, this work pioneers a compartmental Ca^2+^ engineering strategy that unites nanomaterial design, ion signaling, and mechanical immunology, offering a transformative and translatable blueprint for deep PTI against stroma‐rich solid tumors.

## Graphical abstract

A bidirectional calcium‑redistributing nanomodulator rewires intra‑extracellular calcium homeostasis to remodel tumor mechanical microenvironments. It softens tumor stroma to facilitate deep tissue penetration while stiffening tumor cells, evoking amplified immunogenic cell death and robust CTL infiltration, which achieves potent deep photothermal‑immunotherapy against stroma‑rich triple‑negative breast cancer.

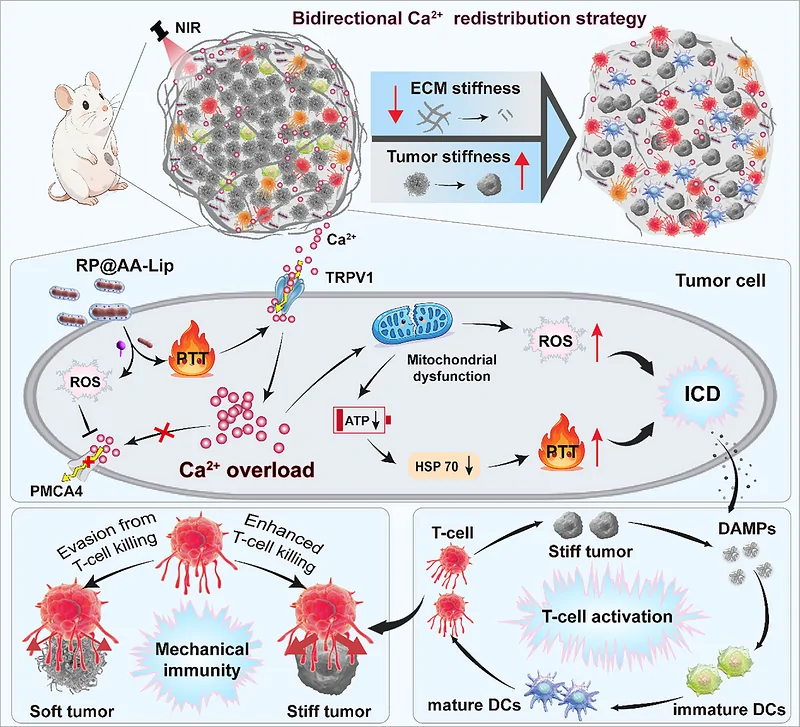

## 1 Introduction

Triple‐negative breast cancer (TNBC) is currently the most execrable type of breast cancer, characterized by rapid proliferation, high metastatic potential, and a notoriously poor prognosis [1, 2]. PTI has emerged as a promising therapeutic modality against TNBC, largely owing to its ability to induce immunogenic cell death (ICD) under near‐infrared (NIR) light exposure, leading to the liberation of damage‐associated molecular patterns (DAMPs) that promote dendritic cells (DCs) maturation and subsequent T cell activation, thereby initiating a systemic antitumor immune response [3, 4, 5, 6, 7]. However, the clinical efficacy of PTI is severely compromised by two intractable barriers inherent to TNBC. A primary obstacle lies in the remarkable adaptive homeostasis of tumor cells, which allows them to engage compensatory survival mechanisms, thereby fostering substantial resistance to therapy and enabling immune evasion [8, 9, 10]. Compounding this issue, the extracellular matrix (ECM) within TNBC lesions is highly dense, containing abundant fibrous proteins, junction proteins, and glycosaminoglycans. Such a physical barrier markedly restricts the entry of photothermal conversion agents (PTCAs) and hampers the infiltration of cytotoxic T cells (CTLs) and other key immune effectors [11, 12, 13, 14]. Given these intertwined difficulties, an ideal strategy for improving PTI outcomes must be able to both dismantle the intrinsic defensive capabilities of cancer cells and remodel the rigid ECM architecture.

Calcium (Ca^2+^) homeostasis plays a fundamental role in driving tumor malignancy, including uncontrolled proliferation, therapeutic resistance, and immunosuppression [15, 16, 17]. Driven by this insight, researchers have developed Ca^2+^ interference‐based treatments. These treatments aim to break the existing calcium homeostasis by exogenously supplementing Ca^2+^ or by emptying internal Ca^2+^ pools, resulting in either an overload or a shortage of intracellular calcium ions [16, 18, 19]. Although such approaches are capable of directly inhibiting tumor proliferation and enhancing cellular susceptibility to other therapeutic modalities, the majority of existing platforms rely on calcium‐containing nanomaterials [20, 21]. This reliance brings about notable safety risks, including acute hypercalcemia, cardiac arrest, and renal failure. By contrast, it may be more favorable to mobilize Ca^2+^ from the tumor's own reserves. That strategy harnesses two key features: the excellent biosafety of the body's own ions, and the steep concentration difference (approximately 10,000–15,000‑fold) between the outside of the cell (1–1.5 × 10^−3^ M) and the inside (≈100 × 10^−9^ M) [22, 23]. It is worth noting that both the structural integrity and the physical stroma of a solid tumor are highly Ca^2+^‐dependent [24, 25]. Tumor cells in a solid mass are bound together by cellular junction proteins, many from the calmodulin family, whose adhesive function requires calcium [24, 26]. Beyond maintaining adhesion, Ca^2+^ also acts as a key mechanical modulator, controlling stromal rigidity and tumor cell stiffness—two parameters that critically govern how well immune cells can infiltrate the tumor and how effectively T cells kill [25, 27]. Based on these facts, we conceptualize a novel strategy named “endogenous Ca^2+^ bidirectional redistribution.” The core idea is to promote Ca^2+^ influx from the extracellular space, leading to a coordinated redistribution of calcium that eventually causes irreversible intracellular calcium overload and extracellular calcium depletion to remodel whole tumor mechanical characteristics.

Tumor cells actually possess a strong Ca^2+^‐buffering system that allows them to quickly offset any calcium influx by activating export pathways [22, 28, 29]. Once cytosolic Ca^2+^ rises, these cells sense the change and promptly pump the excess out through ion channels on the plasma membrane, thereby reducing stress on both the cytoplasm and sensitive organelles like mitochondria [20, 21, 30, 31]. This means that an effective Ca^2^^+^ redistribution strategy must do two things at once: promote calcium entry and block its exit. Two key molecules come into play here—Transient Receptor Potential Vanilloid 1 (TRPV1) and Plasma Membrane Ca^2+^‐ATPase 4 (PMCA4). TRPV1, a Ca^2+^‐permeable channel often overexpressed in TNBC, can be turned on by external triggers such as heat [21, 32]. PMCA4, on the other hand, is an ATP‑driven pump that works to expel Ca^2+^ and keep resting cytosolic levels low [33, 34]. Importantly, when mitochondria are damaged, ATP production drops, which in turn suppresses PMCA4 activity and shuts down this critical export route. Thus, a combination of heat‑triggered TRPV1 activation (opening the inward door) and mitochondrial impairment (closing the outward door) represents a viable strategy for achieving powerful bidirectional Ca^2+^ redistribution.

**SCHEME 1:**
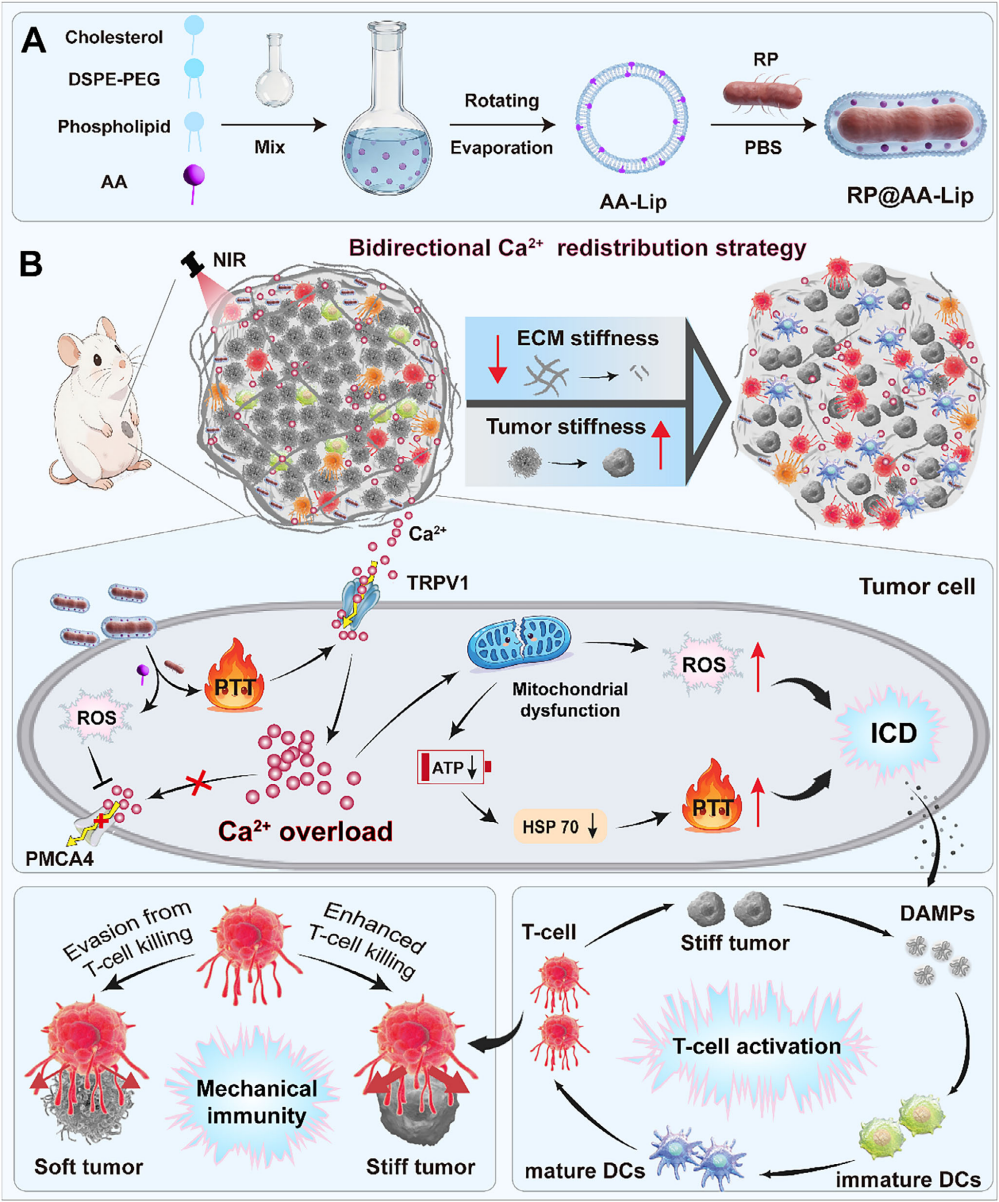
(A) The preparation process of tumor‐targeted RP@AA‐Lip nanomodulator. (B) The mechanism of RP@AA‐Lip‐induced deep PTI for TNBC through a bidirectional Ca^2^^+^ redistribution strategy.

In this study, we have rationally engineered a dual‑channel Ca^2+^ biomodulator, designated RP@AA‐Lip, by coating hypoxia‑targeting Rhodopseudomonas palustris (RP) with an ascorbic acid (AA)‐loaded lipid membrane (Scheme 1A). Following administration, the system autonomously homes in on hypoxic tumor regions and executes a multi‑faceted, coordinated mechanism. Irradiation with NIR light induces local photothermal heating via RP, which in turn activates the TRPV1 channels (often overexpressed in tumors) to drive a substantial influx of Ca^2+^ from the extracellular space. Concurrently, the released AA elevates intracellular ROS levels, causing mitochondrial damage and ATP depletion. This ATP shortfall has two critical consequences: it suppresses PMCA4‑mediated Ca^2+^ efflux and downregulates HSP70, thereby ablating thermoresistance. The outcome is a dramatic and sustained rise in intracellular Ca^2+^, now occurring in cells whose defensive stress responses have been weakened, leading to profound mitochondrial dysfunction and amplified ICD. Meanwhile, the depletion of extracellular Ca^2+^ disrupts cadherin‑dependent cell–cell junctions, loosening the ECM and facilitating both deeper tumor penetration and infiltration by immune cells. At the same time, the intracellular Ca^2^^+^ overload stiffens the tumor cells, establishing a mechanical form of immunosurveillance that improves CTL‑mediated cytotoxicity. In essence, RP@AA‐Lip simultaneously achieves intracellular Ca^2+^ overload and extracellular Ca^2+^ depletion, thereby integrating tumor cell killing, stromal softening, and mechanical immunosurveillance into one coherent therapeutic cascade (Scheme 1B). Distinct from conventional Ca^2+^‑interfering strategies, this tumor‑specific platform synergistically couples bidirectional Ca^2+^ redistribution with energy‑depletion‑driven thermosensitization, presenting a novel paradigm for enhancing deep PTI.

## 2 Results and Discussions

### 2.1 Preparation and Characterization of RP@AA‐Lip

**FIGURE 1:**
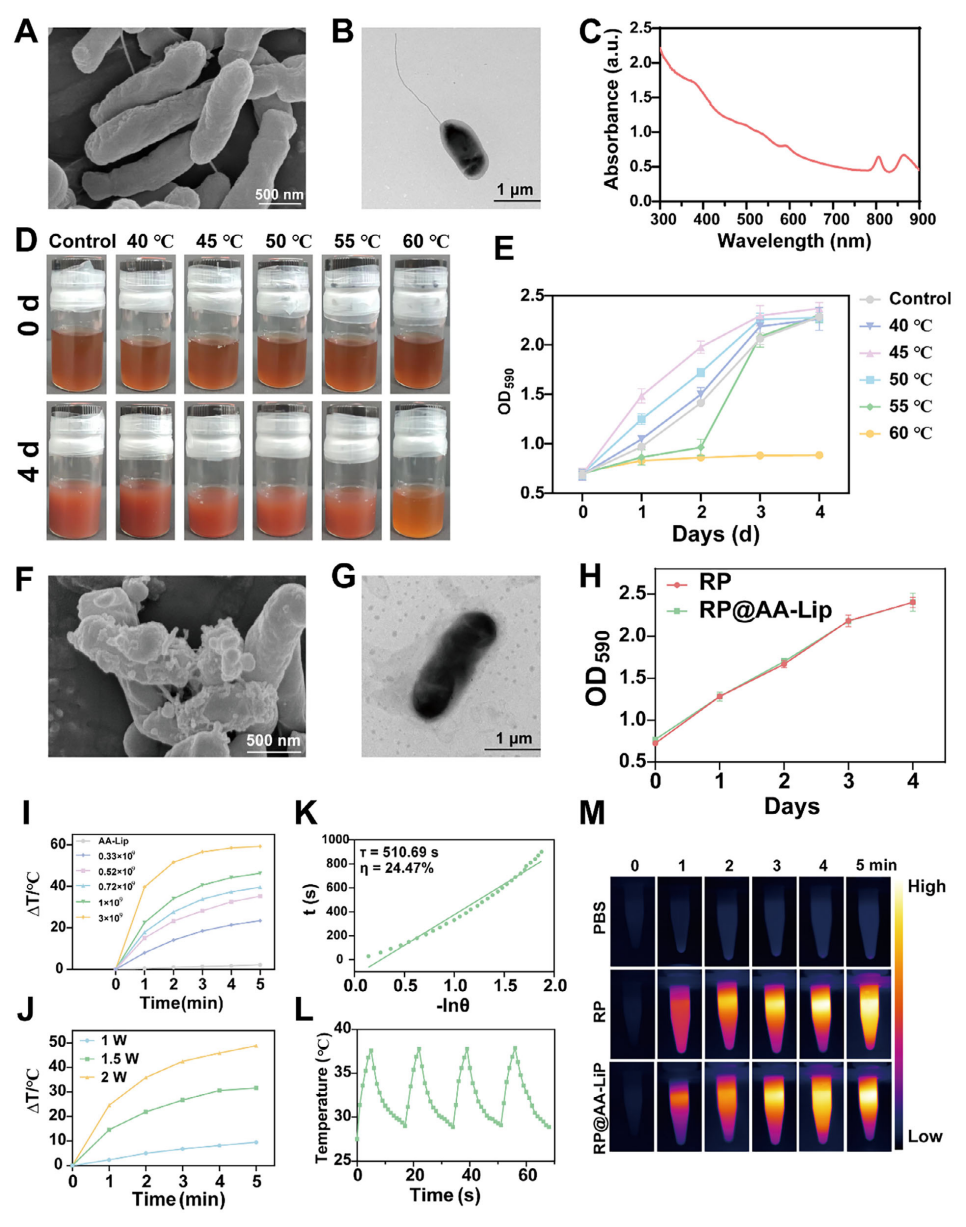
Preparation and characterization of RP@AA‐Lip. (A) SEM image of RP. Scale bar: 500 nm. (B) TEM image of RP. Scale bar: 1 µm. (C) UV–vis–NIR absorption spectra of RP. (D) Photograph of the RP cultured in LB medium after treatment with different temperatures for 30 min. (E) Growth curves of RP cultured in LB medium after treated with different temperatures for 30 min (*n* = 3). (F) SEM image of RP@AA‐Lip. Scale bar: 500 nm. (G) TEM image of RP@AA‐Lip. Scale bar: 1 µm. (H) Growth curves of RP and RP@AA‐Lip (*n* = 3). (I) Concentration‐dependent photothermal heating profiles of RP@AA‐Lip under NIR irradiation (1 W/cm^2^). (J) Photothermal temperature curves of RP@AA‐Lip (RP = 10^9^ CFU/mL) under different 808 nm laser irradiation powers. (K) The photothermal conversion efficiency of RP@AA‐Lip. (L) The photothermal stability of RP@AA‐Lip. (M) Infrared thermal images of RP and RP@AA‐Lip (RP = 10^9^ CFU/mL) after irradiated with NIR (1 W/cm^2^, 5 min).

We cultured the RP and characterized its morphology during the logarithmic growth phase [35, 36]. The morphology of RP was first observed by scanning electron microscopy (SEM) and transmission electron microscopy (TEM). As exhibited in Figure 1A,B, RP displays a typical ovoid shape with a diameter of 1–2 µm, and has flagella at the periphery, which are essential for the motility of the bacteria. The UV–vis–NIR spectra of RP displayed two strong peaks at 808 nm and 850 nm, respectively (Figure 1C), assignable to the characteristic absorptions of bacteriochlorophyll a (BChl a). A critical feature of the photosynthetic bacteria was that they had NIR laser absorption activity, endowing the RP with the ability to convert the NIR energy to heat [37, 38]. Therefore, the photothermal performance of RP was examined using an 808 nm laser. Data from Figure S1A,B show that the solution temperature rose steeply over time with increasing RP concentration or laser power density, demonstrating effective light‑to‑heat conversion. A measured photothermal conversion efficiency of 24.29% (Figure S1C) further attests to this strong performance. Importantly, RP proved highly durable: its heating and cooling profiles did not change appreciably after four on/off irradiation cycles (Figure S1D) and its absorbance remained stable even with extended NIR exposure (Figure S2). Beyond photothermal stability, we also examined bacterial viability. Following four days of incubation at different temperatures, the culture medium retained its dull red hue (Figure 1D) and the OD values at 55°C showed no significant variation (Figure 1E), indicating that RP stays viable throughout the treatment.

Preparation of AA‐enriched lipid membrane by thin film dispersion method and coating it on the RP surface to construct RP@AA‐Lip. First, the AA‐Lip is composed of phospholipid, cholesterol, and DSPE‐PEG mixed with AA. We adopted high‐performance liquid chromatography (HPLC) to quantify AA encapsulation efficiency (EE) and loading content (LC) across varied formulation ratios, with full results summarized in Figure S3. Varying AA feeding doses and lipid blending ratios led to distinct AA loading capacities, mainly governed by cholesterol‐mediated membrane properties [39]. Moderate cholesterol content loosens lipid bilayers and expands internal aqueous microdomains to hold more AA cargo, whereas excess cholesterol compacts the membrane and cuts loading capacity. Additionally, DSPE‐PEG assembles a hydrated outer layer to stabilize liposomal architecture and suppress AA leakage during fabrication. Based on the above formulation screening, we identified the optimal lipid mixing ratio for maximal AA encapsulation. Under this optimized recipe, HPLC quantification further confirmed the total AA loaded onto RP: an average of 105 mg AA was coated onto every 5 × 10^8^ colony‐forming units (CFU) of RP to form the final RP@AA‐Lip biohybrid.

Through TEM and SEM in Figure 1F,G, it can be observed that the surface of RP has become significantly rougher due to a distinct membrane structure around it, confirming the successful preparation of RP@AA‐Lip. Optical density measurements of bacterial colonies showed minimal difference between unmodified RP and the biohybrid RP@AA‐Lip, indicating that the AA‐Lip modification did not compromise bacterial viability (Figure 1H). The colloidal behavior of RP@AA‐Lip in 10% FBS‐containing medium was assessed over 24 and 48 h via dynamic light scattering (DLS). Recorded average hydrodynamic diameters and zeta potentials attested to negligible variations in both size and surface charge throughout the observation window (Figure S4A). Complementing this, a parallel leakage assay revealed that cumulative AA release remained below 15% even after 48 h of incubation under identical serum conditions (Figure S4B). Collectively, these findings substantiate that the lipid bilayer preserves its architectural integrity and resists disruptive effects commonly encountered in protein‐rich environments. We then assessed the photothermal capability of RP@AA‐Lip, as shown in Figure 1I,J. The level of photothermal conversion was clearly dependent on the concentration of RP and the intensity of the laser. Notably, the conversion efficiency of RP@AA‐Lip matched that of naked RP (Figure 1K), indicating that the lipid coating enriched with AA does not compromise the bacterial photothermal function. Repeated on–off cycling (four cycles) produced virtually the same temperature peak each time (Figure 1L), demonstrating that RP@AA‐Lip retains its photothermal stability well. Thermal imaging (Figure 1M) further revealed that both RP and RP@AA‐Lip exhibit substantial heating upon 808 nm laser exposure, while PBS experiences little to no temperature change. Based on these observations, we propose that RP@AA‑Lip is a viable photothermal modulator suitable for PTI‑mediated tumor therapy.

### 2.2 RP@AA‐Lip Mediated Intracellular Ca^2+^ Overload

**FIGURE 2:**
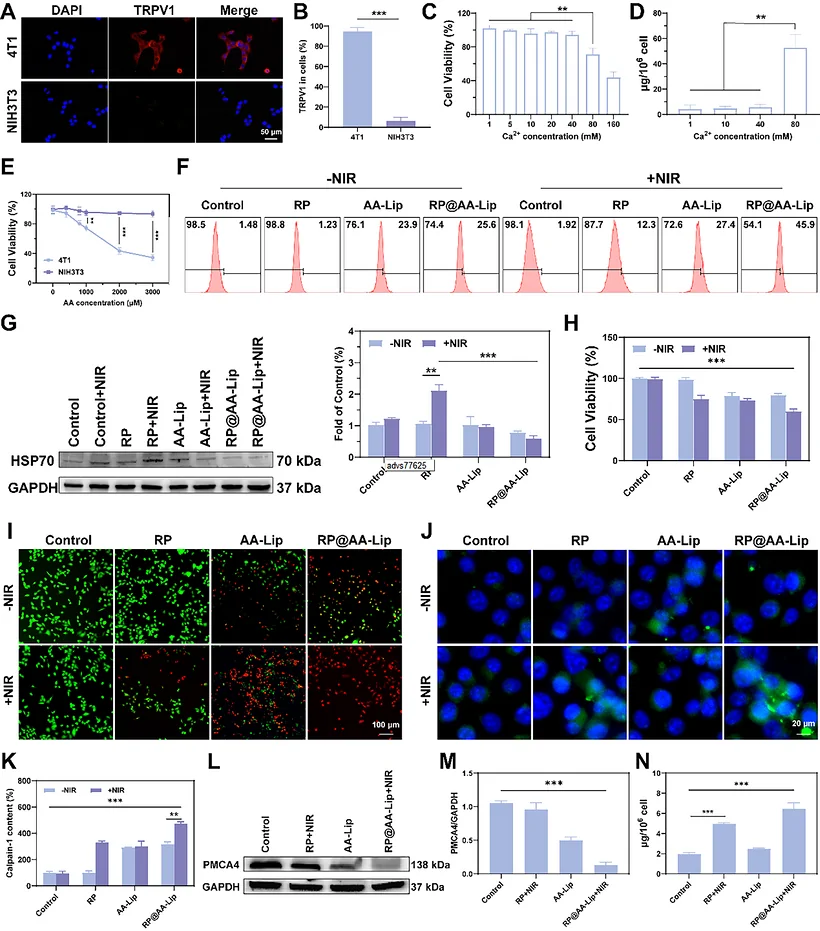
RP@AA‐Lip mediated intracellular Ca^2+^ overload. (A) CLSM images of the TRPV1 expression in 4T1 and NIH3T3 cells. Scale bars: 50 µm. (B) The quantitative analysis of TRPV1 expression in 4T1 and NIH3T3 cells (*n* = 3). (C) Cell viability of Ca^2+^‐treated 4T1 cells was assessed via CCK‐8 assays (*n* = 3). (D) The intracellular Ca^2+^ amount in 4T1 cells with different concentrations of Ca^2+^ treatments was detected using ICP‐MS analysis (*n* = 3). (E) Cytotoxicity of AA against 4T1 and NIH3T3 cells (*n* = 3). (F) Flow cytometry results of ROS exposure levels after PBS, RP, AA‐Lip, or RP@AA‐Lip with/without NIR treatments. (G) Western blot analysis and the corresponding quantitative results of HSP70 expression after PBS, RP, AA‐Lip, or RP@AA‐Lip with/without NIR treatments (*n* = 3). (H) Cell viability after PBS, RP, AA‐Lip, or RP@AA‐Lip with/without NIR treatments (*n* = 3). (I) Fluorescence images of 4T1 cells after PBS, RP, AA‐Lip, or RP@AA‐Lip with/without NIR treatments for live/dead evaluation. Live (Calcein AM, green) and dead (PI, red). Scale bar: 100 µm. (J) CLSM images of 4T1 cells after PBS, RP, AA‐Lip, or RP@AA‐Lip with/without NIR treatment for Ca^2+^ detection. cell nucleus (Hoechst 33342, blue), Ca^2+^ content (Fluo‐4 AM, green). Scale bar: 20 µm. (K) The calpain content in 4T1 cells after indicated treatments was detected using ELISA assay (*n* = 3). (L) Western blot analysis and (M) the corresponding quantitative results of PMCA4 expression after the indicated treatments (*n* = 3). (N) The intracellular Ca^2+^ amount in 4T1 cells after indicated treatments was detected using ICP‐MS analysis (*n* = 3).

TNBC is a highly proliferative and aggressive subtype of breast cancer and exhibits resistance to nearly all therapeutic strategies, including photothermal therapy and immunotherapy [1, 2]. Therefore, we initially selected TNBC as the tumor model to evaluate the therapeutic efficacy of the designed RP@AA‐Lip nanomodulator. Prior to evaluating the biological functions of RP@AA‐Lip, we quantified TRPV1 expression levels in TNBC cells. In agreement with existing literature, TRPV1 was highly expressed in this type of TNBC (Figure 2A). In contrast, TRPV1 protein levels were negligible in the normal tissue‐derived NIH3T3 cell line (Figure 2B). This pronounced cell‐specific overexpression of TRPV1 presents a promising therapeutic target for precise and efficient Ca^2+^ modulation within breast cancer cells. Subsequently, we assessed the capacity of 4T1 breast cancer cells to regulate Ca^2+^ homeostasis. It is remarkable that these cells proliferated normally even when the external Ca^2+^ concentration reached 40 mM (Figure 2C). This value represents a 26.7‐ to 40‐fold increase over the physiological extracellular calcium range of 1–1.5 mM typically observed in the tumor microenvironment. Furthermore, raising the Ca^2+^ concentration from 1 mM to 40 mM did not elicit a detectable rise in intracellular Ca^2+^ in 4T1 cells (Figure 2D), implying an efficient homeostatic mechanism that maintains calcium balance and supports normal cellular activity even under hypercalcemic stress. This regulatory capacity, however, proved insufficient at 80 mM Ca^2+^, where a pronounced Ca^2+^ influx ensued, leading to a marked decline in cell viability.

AA has recently drawn attention not for its nutritional value but for its ability to kill cancer cells [40, 41]. To explore this activity, we treated 4T1 mouse mammary carcinoma cells and non‑malignant NIH3T3 fibroblasts with a series of AA concentrations and assessed viability using the CCK‑8 assay. The results revealed a clear dose‑dependent cytotoxic effect in 4T1 cells, whereas NIH3T3 cells remained largely unaffected across the same concentration range (Figure 2E). It is well established that pharmacologic doses of AA generate reactive oxygen species (ROS) as a primary mechanism of antitumor activity. This ROS production has previously been shown to lower thermal tolerance during PTT by disrupting heat shock protein function. Accordingly, we measured intracellular ROS levels under different treatment conditions. Figure S5 demonstrates that any group containing AA produced bright green fluorescence in 4T1 cells, confirming a strong capacity for ROS generation. In parallel, flow cytometric measurements further corroborated these observations, demonstrating notable ROS buildup in cells treated with any AA‑inclusive preparation (Figure 2F). We then asked whether the accumulated ROS could suppress heat shock proteins and thereby make tumor cells more vulnerable to PTT. Western blot analysis showed that 4T1 cells treated with RP@AA‑Lip plus NIR laser had lower HSP70 levels than cells in other groups, suggesting that this combination indeed enhanced photothermal sensitivity (Figure 2G). In line with this observation, the lowest rate of 4T1 cell viability was found in the RP@AA‑Lip plus NIR group, supporting the idea that AA boosts the tumoricidal efficiency of RP‑mediated PTT (Figure 2H). To distinguish live from dead cells directly, we performed calcein‑AM and propidium iodide (PI) double staining. In the control groups—including the RP‑alone group without NIR exposure and the NIR‑only group—bright green fluorescence was consistently observed, indicating that the vast majority of cells remained viable. In sharp contrast, the RP@AA‑Lip plus NIR group displayed markedly stronger red fluorescence than any other condition, highlighting the potent tumor‑ablative efficacy of this biohybrid system (Figures 2I and S6). Similarly, we performed Annexin V/PI staining to evaluate the cytotoxic effect of the nanomodulator. The resulting flow cytometric analysis demonstrated that the apoptotic proportion in the RP@AA‑Lip group subjected to NIR irradiation amounted to 51.5%, a level that was considerably above that found in the other treatment sets (Figure S7).

Having shown that RP@AA‑Lip generates high ROS levels and potentiates photothermal therapy, we next investigated whether this system also triggers intracellular Ca^2+^ overload. Using a Ca^2+^‑specific fluorescent probe, we observed a pronounced rise in intracellular Ca^2+^ following NIR irradiation in 4T1 cells treated with either RP or RP@AA‑Lip, an effect attributable to photothermal activation of TRPV1 channels that facilitates extracellular calcium influx (Figures 2J and S8). In keeping with the low TRPV1 expression characteristic of this cell line, NIH3T3 fibroblasts exhibited no appreciable changes in Ca^2+^ levels across treatment groups (Figure S9). We next examined proteins that regulate calcium homeostasis. Calpain 1 (CAPN1), a calcium‑dependent cysteine protease, is normally inactive at resting Ca^2^^+^ concentrations but becomes activated when calcium overload occurs [42]. Figure 2K demonstrates that RP@AA‑Lip plus NIR robustly increased CAPN1 expression to about 4.7 times that of the untreated control. Concomitantly, this treatment markedly downregulated the calcium extrusion pump PMCA4 (Figure 2L,M). Collectively, these molecular alterations appeared to underpin the Ca^2+^ overload induced by the combined regimen. Quantitative analysis via inductively coupled plasma mass spectrometry (ICP‑MS) confirmed these observations, revealing a 3.3‑fold increase in intracellular Ca^2+^ in the RP@AA‑Lip + NIR group, versus a 2.5‑fold increase in the RP + NIR group, relative to blank controls (Figure 2N). This synergistic outcome likely arises from a dual mechanism: photothermal heating promotes Ca^2+^ entry from the extracellular space, while AA concurrently suppresses Ca^2+^ efflux, collectively driving the overload.

### 2.3 RP@AA‐Lip Induced Mitochondrial Dysfunction, Enhanced Tumor Rigidity and Intercellular Junction Disruption

**FIGURE 3:**
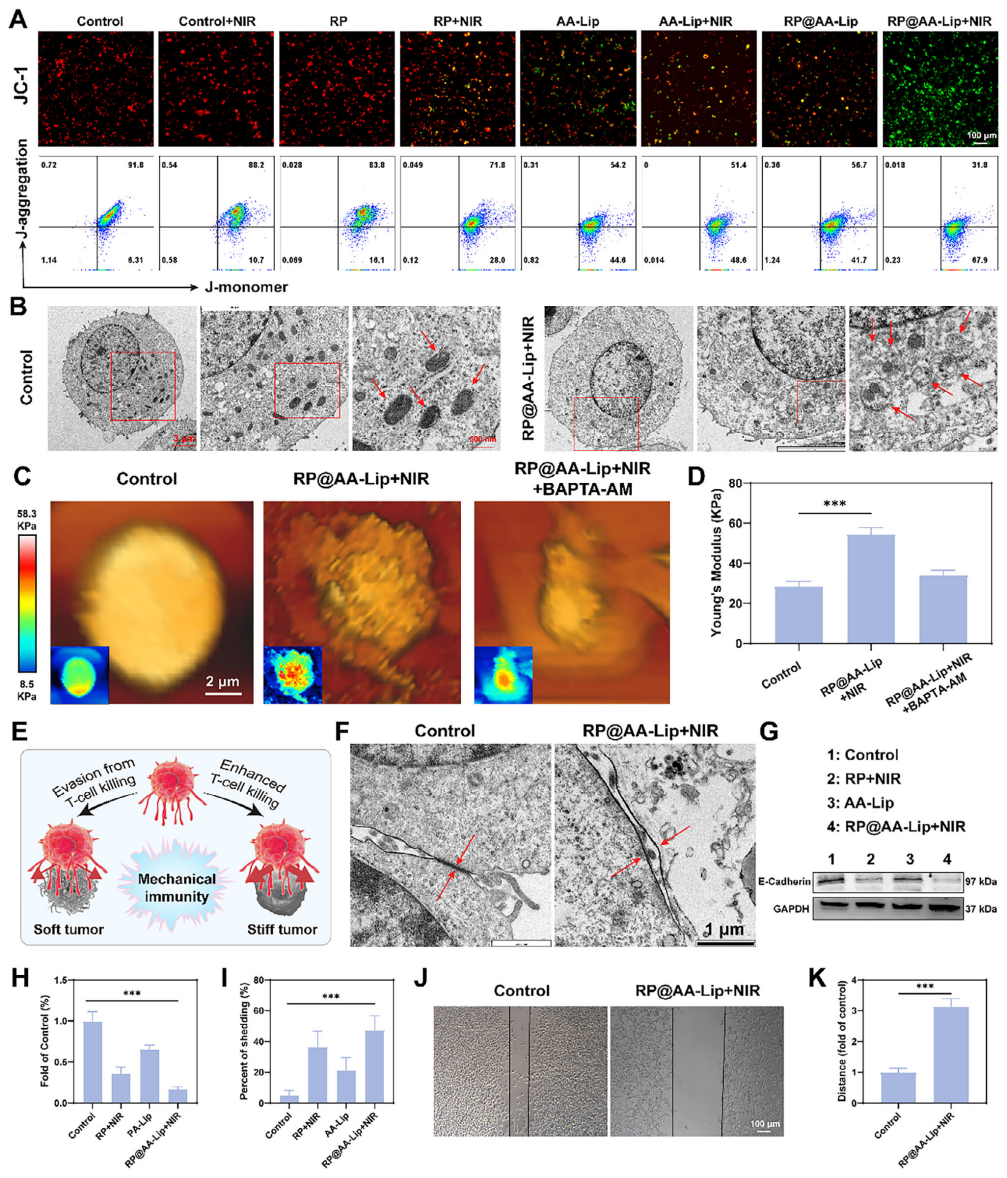
RP@AA‐Lip induced mitochondrial dysfunction and intercellular junction disruption. (A) CLSM images and flow cytometry results of ΔΨm after PBS, RP, AA‐Lip, or RP@AA‐Lip with/without NIR treatments. Scale bar: 100 µm. (B) Bio‐TEM images and the locally enlarged ones of mitochondrial damage within 4T1 cells after different treatments. The red arrows represent the mitochondria. (C) Representative atomic force microscope images of 4T1 cells after PBS, RP@AA‐Lip + NIR, and RP@AA‐Lip + NIR + BAPTA‐PAM treatments. Scale bar: 2 µm. (D) Young's modulus values of 4T1 cells after PBS, RP@AA‐Lip + NIR, and RP@AA‐Lip + NIR + BAPTA treatments (*n* = 3). (E) Schematic illustration of enhanced mechanical immunity. (F) Intercellular junctions of 4T1 cells after indicated treatments were analyzed by Bio‐TEM images. The red arrows represent the cell–cell junctions. (G) Western blot and (H) quantitative analysis of E‐Cadherin of various groups after different treatments (*n* = 3). (I) Percentage of detached cells in suspension detected by flow cytometry (*n* = 3). (J) The wound healing of 4T1 cells after indicated treatments. (K) The quantitation results of migration distances after indicated treatments (*n* = 3).

Recent years have witnessed a surge of research into cellular biomechanics. Malignant cells, in contrast to their normal counterparts, typically display increased deformability or reduced stiffness [43]. This inherent softness of tumor cells can hinder lymphocyte activation, leading to reduced production of potent effector cytokines by these immune cells [44]. Consequently, the mechanical compliance of cancer cells functions as a physical immune checkpoint, granting them resistance to lymphocyte‐mediated cytotoxicity [45]. When Ca^2+^ binds to calmodulin, it activates myosin light chain kinase (MLCK), which then promotes p‑MLC and enhances myosin contraction [27]. In theory, such increased contraction could elevate the elastic modulus of the cell surface. To test this hypothesis, we carried out a series of experiments. We began by measuring p‑MLC expression under various treatment conditions. A clear upregulation of p‑MLC was observed in cells treated with RP@AA‑Lip plus NIR compared to untreated controls (Figure S12). Adding BAPTA‑AM, a calcium chelator, during RP@AA‑Lip plus NIR treatment completely blocked MLC phosphorylation. We then used AFM to directly measure Young's modulus, asking whether disrupted Ca^2+^ signaling influences cell stiffness. RP@AA‑Lip plus NIR‐treated 4T1 cells were found to be 1.9 times stiffer than untreated cells (Figure 3C,D). From a biomechanical standpoint, this change may help tumors escape immune surveillance (Figure 3E). Of note, the stiffness of the RP@AA‑Lip + NIR + BAPTA‑AM group showed no significant difference from the control, implying that the stiffening effect indeed results from an increase in intracellular Ca^2+^.

The calmodulin family of proteins functions as critical intercellular linker molecules that facilitate the aggregation of numerous cancer cells into the densely packed architecture characteristic of tumor tissues, with their binding activity being predominantly regulated by Ca^2+^ [24, 25]. We therefore asked whether the RP@AA‑Lip plus NIR strategy could effectively break down these intratumoral junctions by removing the calcium required for the stability of Ca^2+^‑dependent connexins. Inspection of high‐magnification Bio‐TEM images revealed well‐defined, intact tight junctions between adjacent 4T1 cells in the untreated control group (Figure 3F). In sharp contrast, samples treated with RP@AA‑Lip plus NIR displayed widespread disruption of these intercellular connections. E‐cadherin, often referred to as epithelial cadherin, is a key transmembrane adhesion protein located on the cell surface [24]. It mediates strong cell‑cell binding by forming homophilic bridges with E‑cadherin molecules on neighboring cells, a process fundamentally dependent on Ca^2+^. The western blot analysis revealed a notable reduction in E‑cadherin expression after RP@AA‑Lip plus NIR treatment (Figure 3G,H), suggesting that the lowered extracellular Ca^2+^ compromised E‑cadherin function. When BAPTA‑AM was co‑administered alongside the same treatment, the resultant E‑cadherin signal became statistically indistinguishable from that of the untreated control (Figure S13), further indirectly indicating that the observed reduction in cell‑cell binding is primarily driven by elevated intracellular Ca^2+^ concentration. To quantitatively assess the loss of cell adhesion, we measured the specific rate of cell detachment using flow cytometry (Figure 3I). Untreated control cells remained firmly attached to the substrate, with a detachment rate below 5%. However, within only 6 h after RP@AA‑Lip plus NIR treatment, the desorption rate rose to approximately 45%, providing strong evidence that this treatment promotes cell dissociation. Given that depletion of cellular energy reserves and breakdown of gap junctions would be expected to impair cell motility, we further explored the underlying anti‑metastatic effects. As shown in Figures 3J,K and S14, the migratory and invasive capacities of the cancer cells were significantly suppressed. Following a 24 h incubation with RP@AA‐Lip + NIR, the initially created wound interval in a cell monolayer assay became substantially wider, whereas in the control groups, the gap was largely re‐populated by migrating cells. In summary, these collective findings support the conclusion that the RP@AA‐Lip + NIR treatment paradigm could induce severe mitochondrial dysfunction, enhance tumor rigidity, and cause the disruption of intercellular junctions through a comprehensive redistribution of endogenous Ca^2+^ stores.

### 2.4 Mechanism of RP@AA‐Lip Mediated Cellular Homeostasis Disruption

**FIGURE 4:**
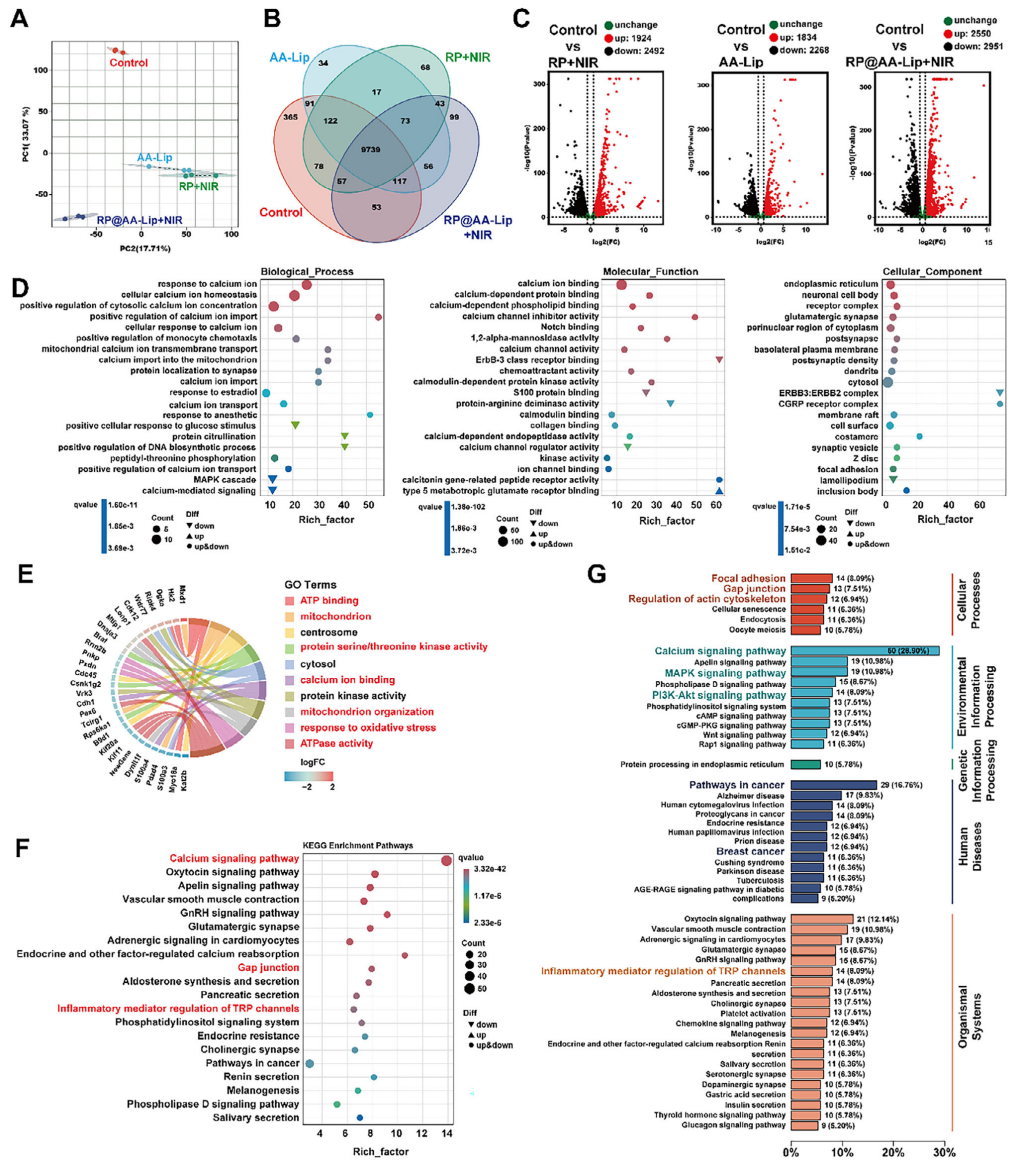
RNA sequencing analysis of RP@AA‐Lip mediated cellular homeostasis disruption. (A) Principal component analysis of each group (*n* = 3). (B) Comparative transcriptome analysis illustrated by a Venn diagram, comparing the control group, RP + NIR group, AA‐Lip group, and RP@AA‐Lip + NIR group (*n* = 3). (C) Volcanic diagram displaying the downregulated and upregulated genes between control group and RP + NIR group, control group and AA‐Lip group, control group and RP@AA‐Lip + NIR group (*n* = 3). (D) GO pathway enrichment analysis for exhibiting the DEGs in the biological process, cellular component, and molecular function between control group and RP@AA‐Lip + NIR group (*n* = 3). (E) GO enrichment analysis chord plot for exhibiting the DEGs in the pathways in 4T1 cells treated with control group and RP@AA‐Lip + NIR group (n = 3). (F) KEGG pathway enrichment analysis for exhibiting the DEGs in 4T1 cells treated with the control group and RP@AA‐Lip + NIR group (*n* = 3). (G) KEGG pathway classification analysis for exhibiting the DEGs in 4T1 cells treated with the control group and RP@AA‐Lip + NIR group (*n* = 3).

Beyond disrupting the function of critical organelles, the accumulation of excessive intracellular Ca^2+^ within tumor cells has the potential to interfere with a wide array of biological pathways that sustain malignant characteristics. To fully characterize the systemic alterations caused by cytosolic Ca^2+^ overload, we conducted RNA‑seq analysis on 4T1 murine breast cancer cells treated with RP@AA‑Lip plus NIR. Principal Component Analysis of the transcriptomic datasets demonstrated high reproducibility across biological replicates within each group and a clear separation between different experimental groups, pointing to distinct gene expression profiles (Figure 4A). A Venn diagram was subsequently prepared to illustrate the overlap and unique DEGs across the comparisons (Figure 4B). Compared to PBS‐treated controls, 4T1 cells exposed to RP plus NIR, AA‑Lip alone, or RP@AA‑Lip plus NIR all showed substantial transcriptomic changes. According to Figures 4C and S15, the numbers of differentially expressed genes were 4416 for RP + NIR, 4102 for AA‑Lip, and 5501 for RP@AA‑Lip + NIR. Among these, the numbers of significantly down‑regulated genes (meeting criteria of fold change < 0.5 and a *p*‐value < 0.05) were 2492, 2268, and 2951, respectively, while significantly up‑regulated genes (fold change > 2 and a *p*‐value < 0.05) numbered 1924, 1834, and 2550, respectively. This remarkable degree of transcriptomic reprogramming emphasizes the powerful effect of calcium overload on intracellular signaling networks.

Subsequent to the identification of DEGs, a comprehensive functional enrichment analysis was performed utilizing the Gene Ontology (GO) knowledge base, with the results visualized in Figures 4D and S16. The data representations, including the enrichment bubble plot and the circular chart diagram, consistently indicated that within the biological process category, the DEGs were predominantly associated with pathways related to response to calcium ion, cellular calcium ion homeostasis, positive regulation of cytosolic calcium ion concentration, and positive regulation of calcium ion import following treatment with RP@AA‐Lip + NIR. For the molecular function classification, significant enrichment was observed for terms encompassing calcium ion binding, calcium‐dependent protein binding, and calmodulin‐dependent protein kinase activity. Regarding cellular component localizations, the analysis identified significant associations with the endoplasmic reticulum, basolateral plasma membrane, cell surface and focal adhesion. A focused examination of the expression patterns of DEGs within these enriched pathways, as depicted in the GO enrichment chord plot, provided further mechanistic insights (Figures 4E and S17). For instance, the upregulation of genes such as Lon peptidase 1 (Lonp1), which is functionally linked to mitochondrial damage and ATPase activity, was noted subsequent to RP@AA‐Lip + NIR exposure. Conversely, a notable downregulation was observed for genes including Cadherin 1 (CDH1), which encodes the E‐cadherin protein, a finding that corroborates the hypothesis that the therapeutic strategy disrupts calcium‐dependent intercellular junctions. Moreover, a predominant suppression was detected in genes implicated in critical DNA repair mechanisms, such as polynucleotide kinase 3'‐phosphatase (PNKP). This transcriptional analysis suggests that the concurrent application of photothermal therapy and the induced high oxidative stress state significantly promoted cell death through synergistic effects, overwhelming the cellular repair capacity.

To further elucidate the underlying molecular mechanisms, a pathway enrichment analysis was conducted using the Kyoto Encyclopedia of Genes and Genomes (KEGG) database. Corroborating the findings from the GO analysis, the KEGG assessment revealed that a significant number of DEGs were prominently enriched in biological processes related to calcium ion transport and the maintenance of cellular calcium homeostasis (Figure 4F). A key finding was the pronounced enrichment of DEGs associated with the inflammatory mediator regulation of TRP channels. This provides strong evidence that the photothermal effect generated by RP serves as a potent activator for these channels, thereby facilitating a substantial influx of extracellular Ca^2+^. Concurrently, the analysis also indicated a notable impact on pathways related to gap junction communication, which is consistent with the observed disruption of intercellular connections likely due to a depletion of local extracellular Ca^2+^. The KEGG classification further identified significant alterations in several other crucial pathways (Figure 4G). These included those governing cell‐matrix and cell‐cell adhesion, such as focal adhesion and gap junction, as well as the regulation of the actin cytoskeleton, which may control tumor stiffness, and the key signaling cascades like the calcium signaling pathway, MAPK signaling pathway, and the PI3K‐Akt signaling pathway. The collective perturbation of these interconnected pathways suggests that the multifunctional RP@AA‐Lip biohybrid, upon NIR irradiation, profoundly disrupts mitochondrial integrity and comprehensively impairs the homeostatic balance within tumor cells.

### 2.5 RP@AA‐Lip Enhances Photothermal‐immunotherapy Through Ca^2+^ Overload

**FIGURE 5:**
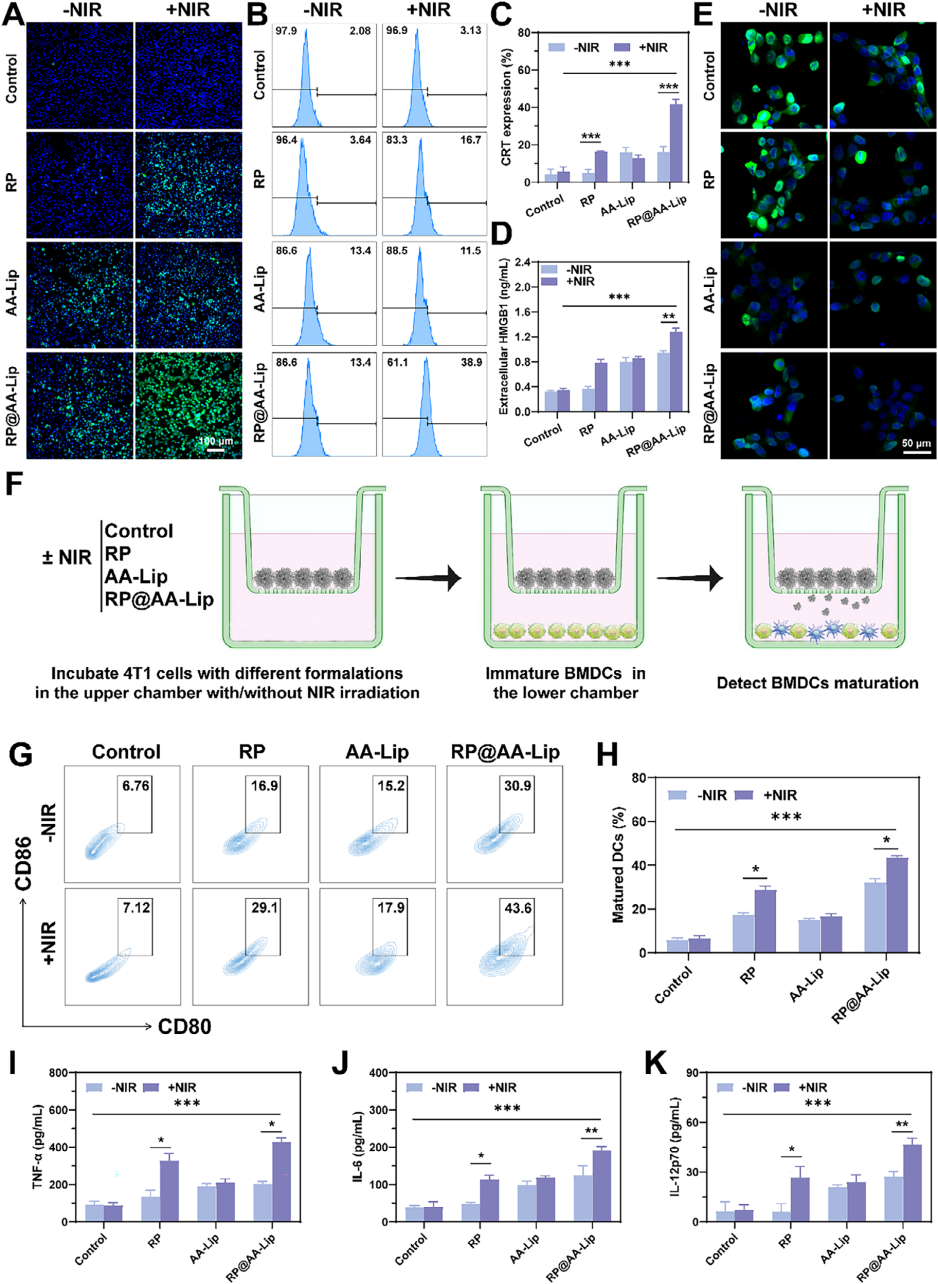
Evaluation of the in vitro immune activation of RP@AA‐Lip. (A) Immunofluorescence images of CRT in 4T1 tumor cells after PBS, RP, AA‐Lip and RP@AA‐Lip treatments with/without NIR irradiation. (B) CRT exposure and (C) the corresponding data were detected by using flow cytometry following PBS, RP, AA‐Lip, and RP@AA‐Lip treatments with/without NIR irradiation (*n* = 3). (D) The quantified data of HMGB1 secretion after PBS, RP, AA‐Lip, or RP@AA‐Lip with/without NIR treatments (*n* = 3). (E) CLSM images of HMGB1 release in 4T1 cells following PBS, RP, AA‐Lip, and RP@AA‐Lip treatments with/without NIR irradiation. (F) Schematic illustration of the experiment of immune cell activation. (G) Flow cytometry and (H) quantification of CD86+CD80+ expression levels on CD11c+ DCs. (I) The supernatant of BMDCs was collected from the indicated groups to detect TNF‐α, (J) IL‐6, and (K) IL‐12p70 cytokine secretion levels (*n* = 3).

PTT, ROS, and Ca^2+^ overload have been established in the literature as potent inducers of ICD within malignant cells [4, 46, 47]. The exposure and release of tumor‐associated antigens and DAMPs during ICD are critical steps that enable the immune system to recognize and eliminate cancer cells. Based on this principle, we examined whether RP@AA‑Lip plus NIR treatment triggers ICD by measuring two well‐recognized biomarkers. One is surface‐exposed calreticulin (CRT), and the other is extracellular high mobility group box 1 protein (HMGB1). Immunofluorescence showed that CRT was prominently translocated to the cell surface in 4T1 cells treated with RP plus NIR, AA‑Lip, or RP@AA‑Lip plus NIR (Figure 5A). In contrast, control groups treated with PBS or RP alone exhibited only minimal CRT exposure. Flow cytometric quantification indicated that the CRT‑positive fraction of tumor cells was markedly elevated to 38.9% following RP@AA‑Lip + NIR exposure, in contrast to the baseline level of 2.08% observed without treatment (Figure 5B,C). We then quantified HMGB1 release into the extracellular space using a specific ELISA. As depicted in Figure 5D, the RP only group without laser irradiation did not show a statistically significant increase in HMGB1 compared to the PBS control. c However, the RP plus NIR, AA‑Lip alone, and RP@AA‑Lip plus NIR groups all showed substantial HMGB1 secretion. Importantly, the extracellular HMGB1 concentration in the RP@AA‑Lip plus NIR group was 1.72 times and 1.65 times greater than that in the RP plus NIR and AA‑Lip groups, respectively. These observations imply that ICD induction depends on either NIR laser exposure or the presence of AA. Meanwhile, the immunofluorescence data (Figure 5E) revealed that RP@AA‑Lip + NIR triggered the most robust HMGB1 egress, thereby indicating its preeminent capacity to stimulate host immunity in the PTI setting. Interestingly, the ICD instigated by this regimen proved to be only partially reversible when BAPTA‑AM was present, failing to achieve complete abrogation (Figure S18). This moderate inhibitory effect is readily explicable, as the immunogenic action of RP@AA‑Lip relies on the synergistic interplay of cytosolic Ca^2+^ overload, thermal cytotoxicity, and ROS generation.

As critical antigen‐presenting cells (APCs), DCs have the essential function of triggering and orchestrating adaptive antitumor immune responses [48]. The process of ICD is known to stimulate the maturation of DCs, which subsequently prime tumor‐specific cytotoxic T lymphocytes to eradicate malignant cells. To investigate the effect of RP@AA‐Lip combined with NIR irradiation on the maturation of DCs, an in vitro transwell co‐culture system was established. In this setup, as schematically represented in Figure 5F, 4T1 cells that had been subjected to the various treatments were seeded in the upper chambers, while bone marrow‐derived dendritic cells (BMDCs) were placed in the lower chambers. Following a 24 h co‐incubation period, the surface level of characteristic costimulatory molecules (CD86 and CD80) on the harvested DCs was measured (Figure 5G). The results from the flow cytometric analysis indicated that the proportion of matured DCs (CD80+CD86+) in the RP + Laser group and the AA‐Lip group was significantly increased, showing 4.29‐fold and 2.57‐fold elevations, respectively, compared to the control group (Figure 5H). We next measured the concentrations of key immune stimulatory cytokines in the collected cell culture supernatants to fully assess the functional activation state of DCs. The secretion levels of interleukin 12p70 (IL‑12p70), tumor necrosis factor α (TNF‑α), and interleukin 6 (IL‑6) were all highest in the supernatant from the RP@AA‑Lip plus NIR group. These concentrations were notably greater than those detected in any other treatment condition (Figure 5I–K). Taken together, these findings support the hypothesis that RP@AA‑Lip, when activated by an 808 nm laser, can induce immunogenic cell death in tumor cells and effectively promote both the maturation and functional activation of DCs. These immunostimulatory properties position this combinatorial approach as a promising candidate for application in photothermal immunotherapy against tumors. Consistent with previous reports that Ca^2+^ overload is a vital ICD trigger, our system achieves synergistic ICD induction through the integration of PTT, ROS accumulation, and Ca^2+^ ‐dependent cytoskeletal and cellular dysfunction. The combination of three independent ICD stimuli produces a mutually reinforced immunogenic death microenvironment, which is superior to single‐factor‐mediated tumor immunogenic injury.

### 2.6 In Vivo Tumor Targeting and Biodistribution of RP@AA‐Lip

**FIGURE 6:**
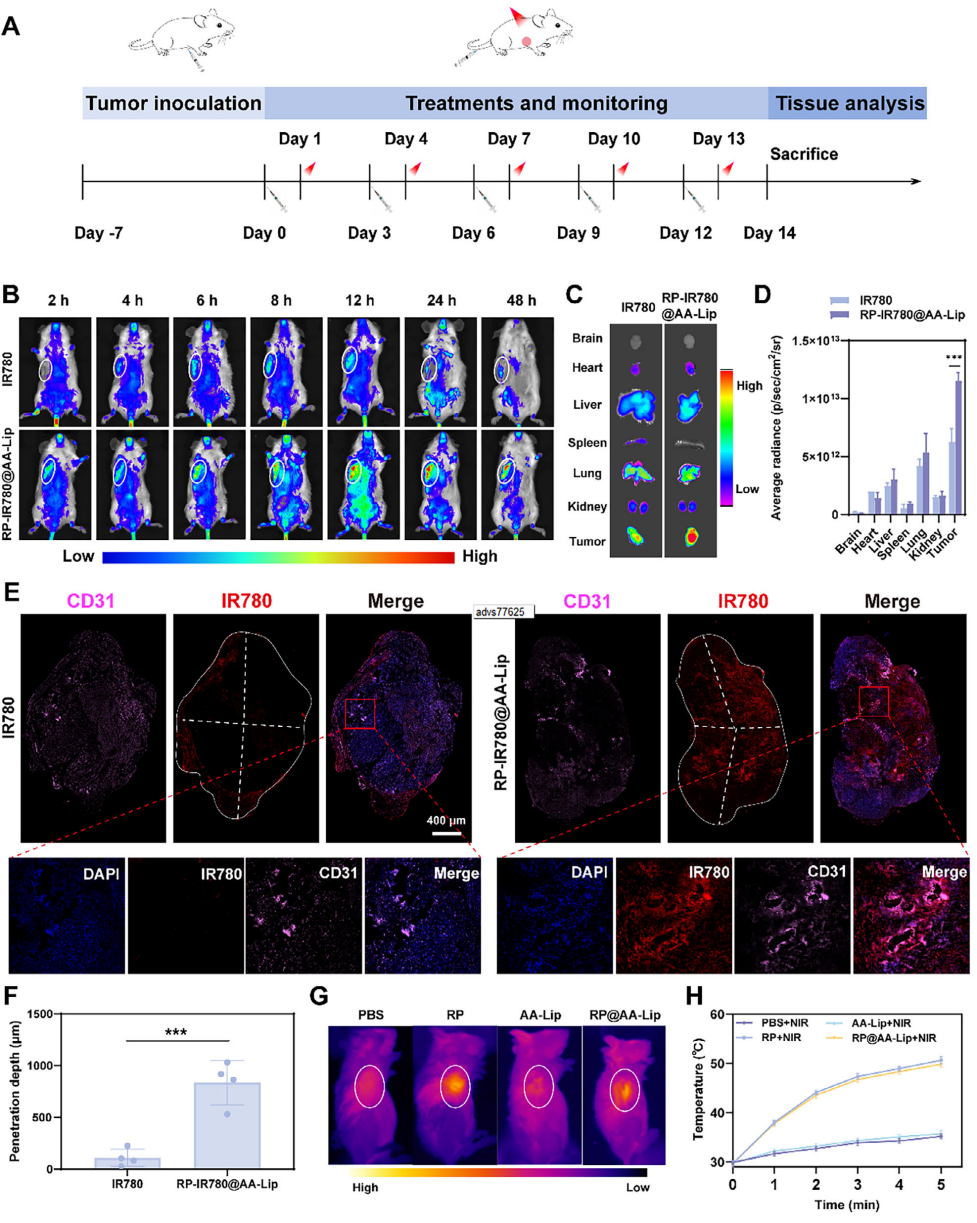
Investigation of the hypoxia‐targeting ability and biodistribution of RP@AA‐Lip. (A) Administration regimen for 4T1 tumor establishment and treatments. (B) In vivo imaging of mice treated with IR780 and RP‐IR780@AA‐Lip. The white circle represents the 4T1 tumor. (C) Comparison of the IR780 fluorescence signals in harvested tumors and major organs following treatment with free IR780 or RP‐IR780@AA‐Lip. (D) Fluorescence quantification maps of resected tumors and organs after administration of IR780 versus the RP‐IR780@AA‐Lip formulation (*n* = 3). (E) Visualization of ex vivo fluorescent images of frozen tumor slices from mice treated with IR780 and RP‐IR780@AA‐Lip. Blue: DAPI; red: IR780; purple: blood vessel. Scale bars: 400 µm. (F) The quantitative tumor penetration depth of IR780 and RP‐IR780@AA‐Lip (*n* = 4). (G) Thermal images of 4T1 tumors with NIR irradiation (1.0 W/cm^2^, 5 min) in mice treated with PBS, RP, AA‐Lip and RP@AA‐Lip. (H) Temperature changes in tumor tissues with NIR irradiation (1.0 W/cm^2^, 5 min) in mice treated with PBS, RP, AA‐Lip and RP@AA‐Lip (*n* = 3).

Building upon the compelling evidence of pronounced mitochondrial disruption and the substantial Ca^2+^ overload‐induced sensitization to photothermal‐immunotherapy achieved by RP@AA‐Lip, we proceeded to evaluate its overall antitumor efficacy in vivo (Figure 6A). Prior to conducting these therapeutic studies, a preliminary investigation into the potential systemic toxicity of the components was undertaken. A suspension of RP at 2×10^9^ CFU/mL was given intravenously to healthy BALB/c mice. Subsequent blood tests showed no statistically significant differences in key hematological parameters between the control group and the RP‐treated animals (Table S1). Meanwhile, peripheral blood plasma C‐reactive protein (CRP) and procalcitonin (PCT) tests showed that no significant elevation of CRP or PCT was observed in RP‑receiving mice relative to the negative control at 24 h and on day 7 (Figure S19). In addition, all mice gained weight steadily and normally throughout the study period, further indicating that the RP component itself caused minimal, if any, systemic toxicity (Table S2). Together, these results support the preliminary safety of using RP as a therapeutic agent in mouse models. Nevertheless, thorough safety evaluations in higher‐order animal species or human subjects will be required before any future clinical translation.

After completing the safety evaluation, we established a murine model bearing 4T1 breast tumors. To examine biodistribution, mice received an intravenous injection of IR780‐labeled RP@AA‑Lip biohybrid, and its localization was tracked non‑invasively at designated time points using an IVIS imaging system. Real‑time in vivo imaging revealed that animals given free IR780 dye showed only a weak fluorescent signal at the tumor site, which faded quickly within 24 h. This finding suggests rapid clearance of the unencapsulated dye from tumor tissues. In contrast, mice treated with RP@AA‑Lip displayed a time‐dependent increase in fluorescence at the tumor location over the same 24‐hour period, indicating improved retention (Figure 6B). Ex vivo imaging of resected tumors and major organs at 48 h confirmed that the RP@AA‑Lip group had a significantly stronger fluorescent signal in tumor tissues relative to other organs (Figure 6C). Quantitative intensity analysis showed that the tumor signal in the RP@AA‑Lip cohort was 1.84‐fold higher than that in the free IR780 group (Figure 6D), unequivocally demonstrating the excellent tumor targeting and accumulation capability of RP@AA‑Lip. This observation is consistent with our in vitro cellular uptake assay under normoxic versus hypoxic conditions, jointly demonstrating the hypoxia‑tumor‑targeting capability of the RP@AA‑Lip biohybrid (Figure S20). To further validate whether RP@AA‑Lip enhances tumor penetration upon NIR irradiation, we prepared frozen sections from tumors collected from mice treated with either free IR780 or RP‑IR780@AA‑Lip and examined them by confocal laser scanning microscopy (CLSM). Blood vessels were visualized by immunofluorescence staining against CD31. As shown in Figure 6E, in the free IR780 group, fluorescent signals were mostly restricted to the tumor periphery and barely detectable in deeper areas. By contrast, the RP@AA‑Lip group exhibited strong red fluorescence that had extravasated from blood vessels and spread evenly throughout the entire tumor region, achieving approximately 7.79‐fold greater penetration depth than the IR780 group (Figure 6F). These observations highlight the superior ability of our biomodulator to promote both accumulation and penetration within tumor tissues.

After validating the preferential tumor accumulation and penetration, we further characterized the in vivo photothermal conversion capability of RP@AA‐Lip via an infrared thermal imaging camera (Figure 6G). The local temperature of the tumor region under irradiation rapidly increased, reaching approximately 50°C within 5 min, a temperature threshold known to induce effective thermal ablation of cancerous cells (Figure 6H). This robust photothermal performance confirms the potential of RP@AA‐Lip for efficient in vivo tumor cell elimination and establishes a solid foundation for its subsequent application in combination therapy.

### 2.7 In Vivo Antitumor Efficacy of RP@AA‐Lip

**FIGURE 7:**
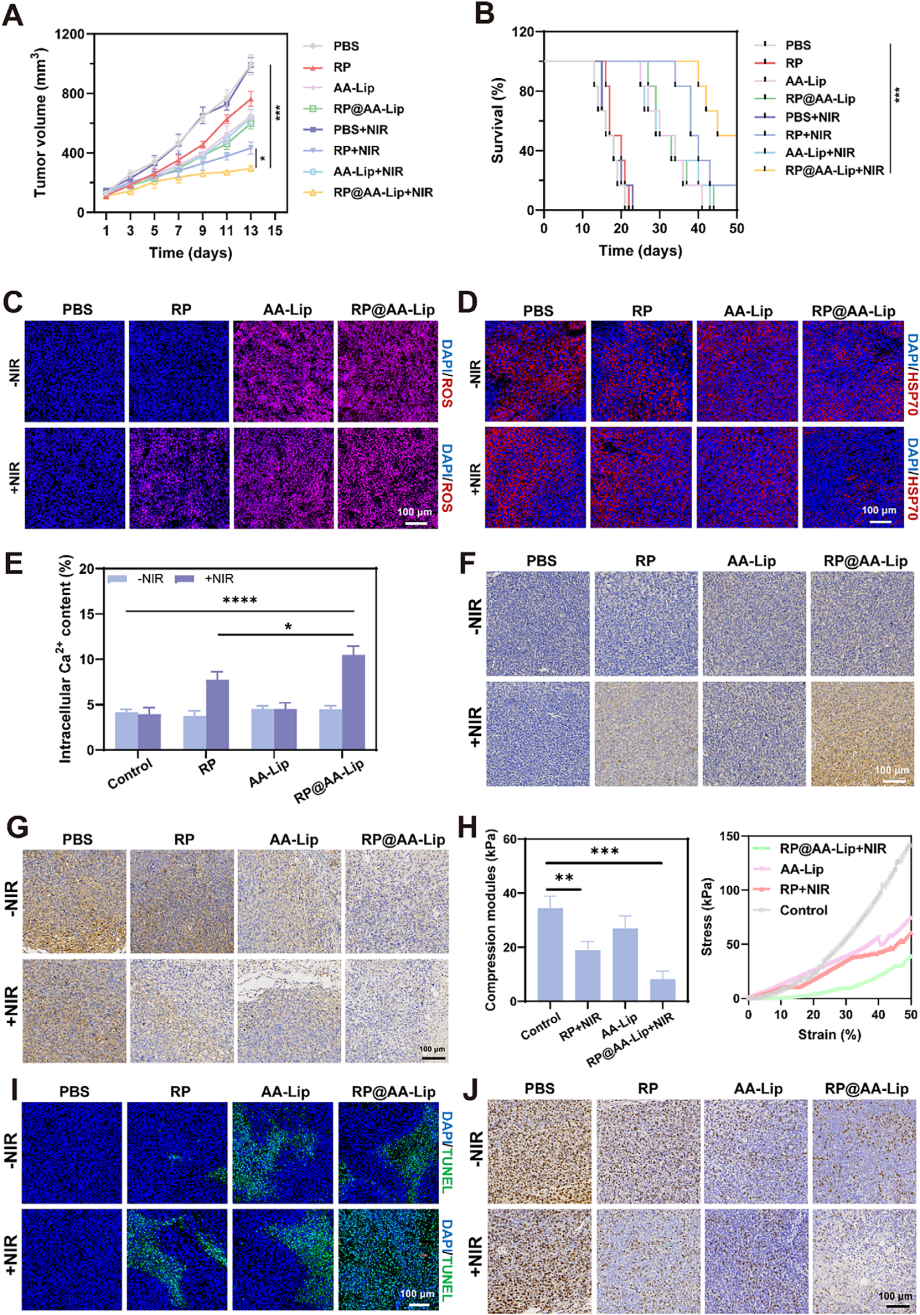
In vivo antitumor efficacy of RP@AA‐Lip. (A) Average tumor growth curves after PBS, RP, AA‐Lip, or RP@AA‐Lip with/without NIR treatments (*n* = 5). (B) Survival curves of mice after PBS, RP, AA‐Lip, or RP@AA‐Lip with/without NIR treatments (*n* = 6). (C) ROS level within the tumor after PBS, RP, AA‐Lip, or RP@AA‐Lip with/without NIR treatments. Scale bar: 100 µm. (D) Hsp70 expression within tumor after PBS, RP, AA‐Lip, or RP@AA‐Lip with/without NIR treatments. Scale bar: 100 µm. (E) The intracellular Ca^2+^ amount in tumors after PBS, RP, AA‐Lip, or RP@AA‐Lip with/without NIR treatments (*n* = 3). (F) CAPN1 staining of tumor tissues after PBS, RP, AA‐Lip, or RP@AA‐Lip with/without NIR treatments. Scale bar: 100 µm. (G) Visualization of E‐Cadherin expression in tumor tissues after PBS, RP, AA‐Lip, or RP@AA‐Lip with/without NIR treatments. Scale bar: 100 µm. (H) The compression moduli and compressive curves evaluating the tumor stiffness after PBS, RP, AA‐Lip, or RP@AA‐Lip with/without NIR irradiation (*n* = 3). (I) TUNEL‐stained tumor sections to evaluate the apoptosis of cells after PBS, RP, AA‐Lip, or RP@AA‐Lip with/without NIR irradiation. Scale bar: 100 µm. (J) Representative Ki67‐stained tumor sections to evaluate the proliferation of cells after PBS, RP, AA‐Lip, or RP@AA‐Lip with/without NIR irradiation. Scale bar: 100 µm.

Considering the encouraging outcomes observed in vitro, where RP@AA‐Lip disrupts cellular homeostasis through Ca^2+^ overload to improve photothermal‐immunotherapy, we postulated that this multifunctional biohybrid would demonstrate enhanced antitumor effectiveness under in vivo conditions. To substantiate this hypothesis, a systematic evaluation of the therapeutic efficacy of RP@AA‐Lip was conducted using a BALB/C mouse model bearing subcutaneous 4T1 tumors, following the experimental timeline detailed in Figure 6A. As illustrated in Figure 7A, monotherapy involving either RP alone or AA‐Lip by itself resulted in only a modest suppression of tumor growth. Conversely, subjects treated with the combination of RP@AA‐Lip and NIR laser irradiation exhibited significantly inhibited tumor progression. At day 14, the mean tumor volume in the combination group measured under 300 mm^3^, a value considerably lower than that seen in any other cohort. According to the survival analysis in Figure 7B, the RP@AA‑Lip plus NIR group also achieved a higher survival rate. Taken together, these findings suggest that the combined regimen enhances both the quality and the duration of survival in mice bearing tumors.

On the 14th day of treatment, mice from each cohort were euthanized so that tumors could be collected for analysis of intracellular ROS and HSP70 levels. Inspection of tumor sections in Figure 7C revealed an intense red fluorescence signal restricted to groups receiving AA‐loaded formulations, indicating that these treatments created a strong oxidative stress state. At the same time, HSP70 measurements provided a crucial observation. The data confirmed that AA in the RP@AA‑Lip + NIR group successfully blocked the HSP70 upregulation normally seen after NIR laser exposure (Figure 7D). We then isolated 4T1 cells from the excised tumors and used ICP‑MS to quantify their Ca^2+^ content (Figure 7E). The highest intracellular Ca^2+^ concentrations were found in the RP plus NIR and RP@AA‑Lip plus NIR groups. In contrast, the RP alone, AA‑Lip alone, and AA‑Lip plus NIR groups showed no significant Ca^2+^ increase. This difference reflects the lack of sufficient photothermal stimulation in those three groups, which is essential for activating TRPV1 and allowing extracellular calcium to enter the cells. Expression patterns of calcium signaling proteins were also evaluated. CAPN1 and E‑cadherin correlated positively with intracellular and extracellular Ca^2+^ concentrations, respectively. Immunoblotting showed that the RP@AA‑Lip + NIR group had a sharp rise in CAPN1 and a steep decline in E‑cadherin (Figure 7F,G). TNBC is characterized by high mechanical stiffness due to its densely packed ECM. To explore how reduced cell junctions affect tissue rigidity, we measured the compressive modulus of the tumors (Figure 7H). By day 8, the compression modulus had decreased to 8.1 kPa in the RP@AA‑Lip + NIR group, compared with 34.3 kPa in the PBS control and 18.9 kPa in the RP plus NIR group (which exhibited a moderate reduction). These results suggest that following RP@AA‑Lip plus NIR treatment, a large amount of Ca^2+^ moves from the intercellular space into the cytoplasm, causing both intracellular Ca^2+^ overload and a notable decrease in tumor matrix stiffness.

A panel of histological methods including H&E staining, TUNEL, and Ki67 staining was employed to comprehensively assess antitumor effects. In the RP@AA‑Lip plus NIR‐treated cohort, H&E‐stained tumor sections exhibited the most widespread regions without cellular nuclei, reflecting the greatest degree of cell death among all groups (Figure S21). TUNEL staining, which labels apoptotic DNA fragmentation, gave the brightest fluorescence in this same group, thereby confirming maximal apoptotic activity (Figure 7I). Ki67 immunostaining, a measure of cell proliferation, showed that the RP@AA‑Lip plus NIR group had a markedly lower fraction of proliferating tumor cells than groups receiving AA‑Lip alone or phototherapy alone (Figure 7J). These histological results are consistent with the tumor growth inhibition data.

In light of the fact that RP@AA‐Lip is a biohybrid agent derived from bacterial components and designed for photothermal‐immunotherapy, a thorough assessment of its biosafety profile was undertaken. This involved monitoring changes in body weight and conducting histological analysis of isolated tissues across all groups. The results showed hardly any variations in body weight and no detectable pathological damage in the harvested organs from treated mice compared to the control group (Figures S22 and S23). To further investigate systemic toxicity, blood biochemistry analyses were performed to assess hepatic and renal functions (Figure S24). All measured parameters related to liver and kidney health remained within normal physiological ranges. Taken together, the data substantiate the conclusion that RP@AA‐Lip constitutes a safe and highly effective system for conducting antitumor photothermal‐immunotherapy under in vivo conditions.

### 2.8 In Vivo Immune Activation of RP@AA‐Lip

**FIGURE 8:**
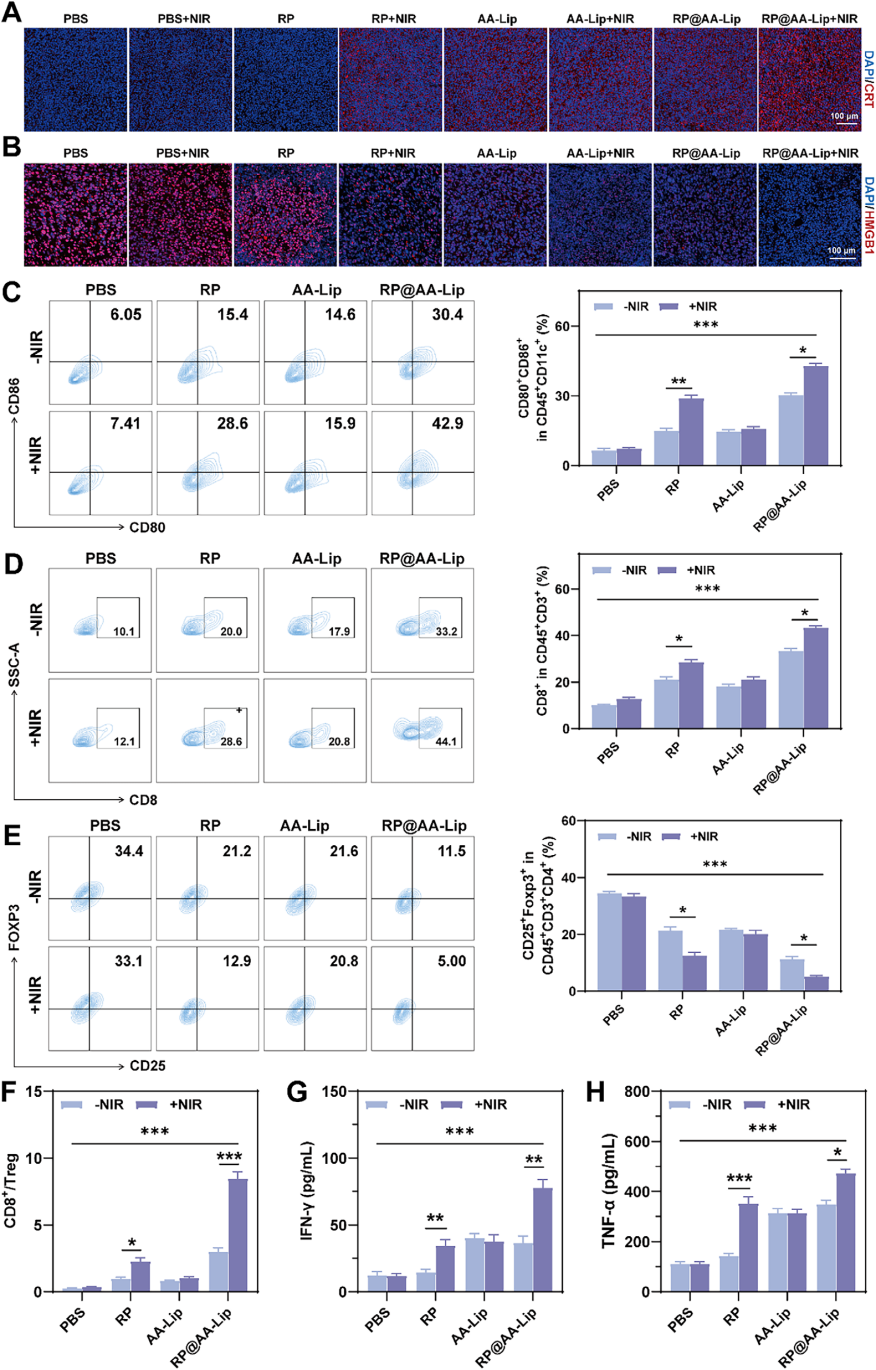
In vivo anti‐tumor immunity of RP@AA‐Lip. (A) Expression of CRT within the tumor after PBS, RP, AA‐Lip, or RP@AA‐Lip with/without NIR exposure. Scale bar: 100 µm. (B) Immunofluorescence staining of HMGB1 within the tumor in PBS, RP, AA‐Lip, or RP@AA‐Lip groups with/without NIR exposure. Scale bar: 100 µm. (C) The representative flow cytometry histogram and the corresponding quantitative data of mature DCs within the tumor after PBS, RP, AA‐Lip, or RP@AA‐Lip with/without NIR treatments (*n* = 3). (D) Flow cytometry histogram and the corresponding quantitative data of CD3+CD8+ CTLs within the tumor in PBS, RP, AA‐Lip, or RP@AA‐Lip groups with/without NIR exposure (*n* = 3). (E) Flow cytometry histogram and the corresponding quantitative data of Tregs within the tumor after PBS, RP, AA‐Lip, or RP@AA‐Lip with/without NIR treatments (*n* = 3). (F) The CD8+ T cells to Tregs ratios in the tumor after various treatments (*n* = 3). (G) The concentration of IFN‐γ and (H) TNF‐α in serum from mice after various treatments (*n* = 3).

To investigate the underlying antitumor immune mechanisms activated by RP@AA‐Lip, we conducted a systematic analysis of tumor‐infiltrating immune cell populations and cytokine secretion profiles following three consecutive treatment cycles. Immunofluorescence examination of tumor sections, as presented in Figure 8A,B, revealed substantially elevated CRT expression and HMGB1 release in the RP@AA‐Lip + NIR group relative to all other experimental groups. This pronounced biomarker expression provides compelling evidence for a robust ICD response occurring under in vivo conditions. We then assessed DC maturation status by flow cytometric measurement of CD11c+CD86+CD80+ cell populations within tumor‑draining lymph nodes. The data showed that mature CD80+CD86+ DC frequencies in TDLNs rose by 4.15‑fold in the RP plus NIR group and by 6.22‑fold in the RP@AA‑Lip plus NIR group relative to the control (Figures 8C and S25). Such enhancement in DC maturation probably results from the strong release of tumor antigens consequent to Ca^2+^ overload augmented by photothermal immunotherapy.

A key limitation of immunotherapy in TNBC is the immunosuppressive tumor microenvironment, which typically shows scarce CTL infiltration and abundant Tregs. We therefore investigated CTL accumulation within tumors after RP@AA‑Lip + NIR treatment, which softens the tumor matrix and enhances the penetration of the biomodulator. Flow cytometric analysis revealed that the RP@AA‑Lip + NIR group exhibited the highest CD3+CD8+ CTL level among all cohorts, representing a 4.45‐fold increase over the control (Figure 8D). An analogous expansion of CTLs was seen in the spleens of the same treatment group (Figure S26), indicating that the enhanced combination therapy elicited a systemic antitumor T cell response. Given that Tregs can suppress CTL function in breast cancer, we also assessed intratumoral CD3+CD4+CD25+FOXP3+ Treg frequencies. The data showed that RP@AA‑Lip plus NIR therapy lowered Treg infiltration by 29.8% compared to the control group (Figures 8E and S27). Consequently, the CD8+ CTL to Treg ratio rose markedly in the combination therapy group, with 3.5‐fold and 9.6‐fold increases over the RP plus NIR and AA‑Lip monotherapy groups, respectively, reflecting a beneficial change in the immune environment (Figure 8F). To further support these cellular findings, we measured systemic cytokine levels in serum collected from all experimental animals. ELISA results indicated that mice treated with RP@AA‑Lip plus NIR had serum IFN‑γ and TNF‑α levels that were 2.6‐fold and 3.3‐fold higher, respectively, than those in control mice (Figure 8G,H). Collectively, these multifaceted immunological assessments demonstrate that the Ca^2+^ overload‐potentiated photothermal‐immunotherapy effectively enhances antitumor T cell immunity and promotes comprehensive remodeling of the tumor microenvironment, ultimately resulting in superior tumor suppression.

## 3 Conclusion

In summary, we engineered a Ca^2+^‐free biomodulator capable of co‐targeting TRPV1 and PMCA4 channels, thereby triggering concurrent intracellular Ca^2+^ overload and extracellular Ca^2+^ depletion to potentiate PTI. This rise in cytosolic Ca^2+^ severely compromised cellular homeostasis, as indicated by impaired organelle function, improved tumor stiffness, and widespread transcriptomic alterations. Furthermore, depleting Ca^2+^ from calcium‐dependent connexins degraded intercellular junctions within tumors, promoting tissue loosening and enhancing deep penetration of the biomodulator. In vivo, the hypoxia‐targeted RP@AA‐Lip accumulated preferentially in tumor regions, enhancing the penetration of the biomodulator, intensifying immunogenic cell death, promoting dendritic cell maturation and T cell activation, stimulating cytokine release, and limiting regulatory T cell infiltration, which collectively amplified the antitumor effect of PTI in TNBC models. These results clarify the role of Ca^2+^‐mediated disruption of cellular homeostasis in disorder‐based therapy and establish a strategy for augmenting PTI outcomes in treatment tumors via endogenous Ca^2+^ bidirectional redistribution and tumor mechanical remodeling.

## Author Contributions

**Hongjuan Zhao**: conceptualization, supervision, funding acquisition, writing – original draft, resources. **Jinpei Sun**: project administration, methodology. **Chenxi Zhao**: methodology, project administration. **Yuting Cang**: validation, visualization. **Keyu Zong**: investigation, formal analysis. **Yuxin Guo**: project administration, formal analysis, methodology. **Yajing Wang**: software, data curation. **Di Meng**: investigation, formal analysis. **Qingling Song**: resources, conceptualization, writing – review and editing, funding acquisition. **Lei Wang**: conceptualization, supervision, resources, writing – review and editing, funding acquisition.

## Conflicts of Interest

The authors declare no conflicts of interest.

## Supporting information




**Supporting File**: advs77625‐sup‐0001‐SuppMat.docx.

## Data Availability

The data that support the findings of this study are available from the corresponding author upon reasonable request.
