# Supplementary material for: Tumor Mechanical Remodeling via Bidirectional Calcium Redistribution Enables Deep Photothermal‐Immunotherapy
Source: Adv Sci (Weinh). 2026 Sep 8:e77625. Online ahead of print. doi: 10.1002/advs.77625 (PMC13554381; doi:10.1002/advs.77625)
Supplement: Supplementary file 1 [file ADVS-9999-e77625-s001.docx]

**Tumor Mechanical Remodeling via Bidirectional Calcium Redistribution Enables Deep Photothermal-Immunotherapy**

Hongjuan Zhao^1,2,3^, Jinpei Sun^1^, Chenxi Zhao^1^, Yuting Cang^1^, Keyu Zong^1^, Yuxin Guo^1^, Yajing Wang^1^, Di Meng^1^, Qingling Song^1,2, 3^*, Lei Wang^1,2,3^*

1 School of Pharmaceutical Sciences, Zhengzhou University, 100 science avenue, Zhengzhou 450001, China

2 Key Laboratory of Nanomedicine for Targeting Diagnosis and Treatment, Ministry of Education of China, Zhengzhou University, 100 science avenue, Zhengzhou 450001, China

3 School of Pharmaceutical Sciences, State Key Laboratory of Antiviral Drugs, Pingyuan Laboratory, Zhengzhou University, Zhengzhou 450001, China

*Corresponding author email: [wanglei1@zzu.edu.cn](mailto:wanglei1@zzu.edu.cn); [songqingling@zzu.edu.cn](mailto:songqingling@zzu.edu.cn)

**Materials:** Phospholipids, cholesterol (10 mg), ascorbyl palmitate (AA), and DSPE-PEG_2000_ were bought from Sigma Aldrich. CCK-8, Calcein-AM and PI live/dead double stain kit, Fluo-4 AM, reactive oxygen species assay kit, calpain assay kit, mitochondrial membrane potential assay kit with JC-1 and ATP assay kit from Beyotime (Shanghai, China). HMGB1, TNF-α, IL-6, IL-12p70 and IFN-γ cytokine ELISA kit were purchased from Peprotech and Enzyme-linked Biotechnology Co., Ltd. (Shanghai, China). Anti-TRPV1, anti-HSP70, anti-PMCA4, and anti-E-Cadherin antibodies were purchased from Abcam and Cell Signaling Technology. Roswell Park memorial institute-1640 (RPMI 1640) and Dulbecco’s modified Eagle’s medium (DMEM) were obtained from Beijing Solarbio Science & Technology Co., Ltd. (Beijing, China). Fetal bovine serum (FBS) and penicillin-streptomycin were purchased from Gibco (Shanghai, China).

**Bacteria culture:** *Rhodopseudomonas palustris* was obtained from the Guangdong microbial Culture Collection Center (Guangzhou, China). For bacterial culture, RP strains were grown under anaerobic conditions in liquid medium at 30 °C for a duration of 4 days.

**RP@AA-Lip preparation and characterization:** First, phospholipids (70 mg), cholesterol (5, 10 and 20 mg), DSPE-PEG_2000_ (5 mg), and ascorbyl palmitate (AA) at different mass (125, 250 and 500 mg) were separately weighed into pear-shaped flasks. Then, 15 mL of chloroform was added to dissolve the mixture thoroughly, and the chloroform was removed by rotary evaporation, forming a thin film at the bottom of the flask. Subsequently, 10 mL of RP solution (1×10^8^ CFU/mL) was added, and the mixture was incubated by rotary evaporation in a 45 ℃ water bath for 1 h. After incubation, the solution was transferred to a dialysis bag and dialyzed for 12 h to obtain RP@AA-Lip, which was stored at 4 ℃ for future use. TEM (HT7700, Japan) and SEM (SU8020, Japan) were employed to observe the morphology of RP and RP@AA-Lip. A UV–visible-NIR spectrophotometer (Shimadzu, UV-2550) was used to obtain the UV–vis-NIR absorption spectra.

**Quantification of AA encapsulation efficiency (EE) and loading content (LC) via HPLC:** High-performance liquid chromatography (HPLC) was used to quantify unencapsulated AA. The Calculation formulas as following: Encapsulation efficiency (EE, %) = (Total feeding AA mass − Free AA mass in supernatant) / Total feeding AA mass × 100%; Loading content (LC, %) = (Mass of encapsulated AA) / Total mass of RP@AA-Lip nanoparticles × 100%.

HPLC analysis was performed on a C18 column (octadecylsilane‑bonded silica gel as stationary phase) with mobile phase A: KH_2_PO_4_‑methanol (V/V=25/75,), and mobile phase B: tetrahydrofuran‑H_2_O (V/V=45/55). Gradient elution was carried out as follows: 0–30 min, 95 % A; 31–41 min, 5 % A; 42–46 min, 100 % A. The column temperature: 35 ± 5 ◦C. The flow rate was set at 0.7 mL/min and UV detector set as 265 nm.

**RP activity evaluation:** RP and RP@AA-Lip (containing equivalent bacterial loads) were inoculated into culture tubes with liquid medium and cultured under standard conditions for 4 days. The optical density at 590 nm (OD_590_) of bacterial cultures was measured daily to monitor growth kinetics.

**Temperature tolerance assessment:** Temperature tolerance was assessed by harvesting RP via centrifugation and reconstituting them in fresh medium at a density of 1×10^9^ CFU/mL. The bacterial suspensions were then subjected to a 30-min heat challenge at varying temperatures (40, 45, 50, 55, and 60 °C). Following thermal treatment, the samples were inoculated into growth medium and incubated at 30 °C for 4 days. A daily measurement of the optical density at 590 nm (OD_590_) was conducted to evaluate bacterial viability.

**Long-term irradiation tolerance assessment:** To assay NIR irradiation tolerance, RP cells were collected by centrifugation and adjusted to a concentration of 1×10^9^ CFU/mL. Aliquots of this suspension were put through serial exposures for 5, 10, 15 and 20 min under NIR irradiation (CNI, Changchun, China) at a density of 1.0 W/cm^2^. After exposure, the absorbance of RP was measured using ultraviolet spectroscopy (Shimadzu, Japan).

**Photothermal performance assessment:** A series of bacterial suspensions with concentrations ranging from 0.33×10^9^ to 3×10^9^ CFU/mL were prepared for photothermal testing. Aliquots of each suspension were subjected to irradiation from an 808 nm laser source at a fixed power density of 2.0 W/cm^2^ for a duration of 5 min, with PBS serving as a negative control. The resulting temperature profiles were captured at one-minute intervals using an infrared thermal imager (FLUKE, Shanghai, China). Subsequently, the photothermal response of RP (at 1×10^9^ CFU/mL) was investigated under varying power densities of 1.0, 1.5, and 2.0 W/cm^2^, with the same 5-min irradiation period. To evaluate photostability, an identical bacterial suspension was subjected to four cycles of NIR laser exposure (1.0 W/cm^2^, 5 min ON), with intervening cooling phases, while the thermal response was meticulously recorded. Finally, the photothermal conversion efficiency (η) was determined. For this calculation, 1 mL of the bacterial suspension was irradiated with the 808 nm laser at 1.0 W/cm^2^, and real-time temperature data was collected via the thermal camera for application in Equation (1)

$\eta=\frac{hS\left( T_{max}-T_{surr} \right)-Q_{dis}}{I(1-{10}^{-A_{\lambda}})}$	（1）

where h is the heat transfer coefficient, S is the irradiated area, T_max_ represents the maximum steady-state temperature, T_sur_ is the ambient temperature of the surrounding, I is the laser power and A_λ_ is the absorbance of RP at 808 nm.

The steps for evaluating the photothermal performance of RP@AA-Lip are the same as the experimental steps mentioned above.

**Cell culture:** The 4T1 murine breast cancer and NIH3T3 murine fibroblast cell lines were sourced from the Cell Bank of the Chinese Academy of Sciences (Shanghai, China). 4T1 cells were cultured in RPMI 1640 medium and NIH3T3 cells were cultured in DMEM medium supplemented with 10% (v/v) fetal bovine serum and 1% (v/v) antibiotics, respectively. Cells were cultured in an incubator at 37 ℃ in an atmosphere of 5% (v/v) CO_2_.

**TRPV1 detection:** Immunofluorescence staining was employed to determine the expression of TRPV1. 4T1 and NIH3T3 cell lines were plated on confocal dishes at 2 × 10^5^ cells per dish and grown for 24 h at 37 °C. The subsequent steps involved washing the cells with PBS, fixation with 4% paraformaldehyde, permeabilization using chilled methanol, and blocking with 1% BSA. Thereafter, samples were treated overnight at 4 °C with a primary antibody specific to TRPV1, followed by a one-hour incubation with a fluorophore-conjugated secondary antibody at room temperature. Cell nuclei were counterstained with DAPI. Imaging was ultimately performed with a confocal laser scanning microscope (CLSM, Leica, Germany).

**Ca^2+^ homeostasis evaluation:** To investigate calcium homeostasis, 4T1 cells were plated and allowed to adhere for 24 hours. In the initial experiment, cells in 96-well plates were exposed to a serially diluted calcium solution (1-160 μΜ) for 24 hours. Post-incubation, cellular proliferation was evaluated by adding CCK-8 solution and measuring the resulting absorbance at 450 nm after a 2-hour period using a microplate reader (Bio Tek, America). Parallel to this, cells were cultured in 6-well plates and treated with specific calcium concentrations (1, 10, 40, 80 μΜ) for 8 hours. Upon completion of the treatment interval, these cells were trypsinized, counted, and prepared for inductively coupled plasma mass spectrometry (ICP-MS, PE, Nexlon 2000) analysis to determine intracellular calcium content.

**Cell cytotoxicity assay:** The in vitro cytotoxicity of AA against 4T1 and NIH3T3 cells was evaluated using the CCK-8 assay. Cells were seeded in a 96-well plate and incubated for 24 h, a serially diluted AA solution (0-3000 μΜ) was added. After an additional 24 h incubation period, the medium was removed from each well, and the adherent cells were subjected to three washes with PBS. Subsequently, a fresh mixture consisting of 100 μL of culture medium and 10 μL of CCK-8 solution was introduced into the wells. The plate was then allowed to incubate for a further 3 h, after which the absorbance was read at a wavelength of 450 nm.

**Live/Dead cell staining assay:** A Calcein-AM/PI dual staining assay and cell apoptosis assay were also employed to evaluate the antitumor effects of the indicated therapies against 4T1 cells. Cells were seeded into a 12-well plate and cultured for 24 h, after which Ca^2+^ (40 mΜ)-contained medium involving PBS, RP (2 × 10^9^ CFU/mL), AA-Lip (1.0 mM), or RP@AA-Lip was added. After 3 h incubation, the samples designated for the NIR groups were exposed to an 808 nm laser at a power density of 1.0 W/cm^2^ for 5 min. Upon completion of a further 24-hour incubation, the supernatant in the wells was discarded and the cells were carefully washed three times using PBS. For Calcein-AM/PI dual staining assay, the cells were subjected to a 30 min staining procedure at 37 °C using Calcein-AM and PI to distinguish live and dead populations. Fluorescence images were finally acquired by CLSM. For cell apoptosis assay, the cells were staining with Annexin V‑FITC/PI following test instructions and detected using flow cytometry.

**Ca^2+^ channel activity assay:** Ca^2+^ channel activity was detected through. Cells were inoculated into a culture dish and incubated for 24 h. Then, the culture medium was replaced with fresh Ca^2+^ (40 mΜ)-contained medium involving PBS, RP (1 × 10^9^ CFU/mL), AA-Lip (1.0 mM), or RP@AA-Lip. The NIR groups were incubated for 3 h and then irradiated with a NIR laser (1.0 W/cm^2^, 5 min). Following an additional 6 h incubation, cells were harvested for analysis of intracellular Calpain and PMCA4. For PMCA4 detection, protein extracts were prepared from lysed cells. Following separation on a 10% SDS-PAGE gel and subsequent transfer to a PVDF membrane, the extracted proteins were analyzed by immunoblotting. The membrane was first probed with an anti-PMCA4 primary antibody overnight at 4 °C. After three wash cycles, a one-hour incubation with a suitable HRP-linked secondary antibody was performed at room temperature. The target protein bands were finally visualized via ECL luminescence. For Calpain activity assessment, cell lysates were centrifuged, and the resulting supernatant was analyzed according to the manufacturer’s protocol for calpain activity.

**Determination of intracellular Ca^2+^ level:** Intracellular Ca^2+^ levels were assessed using both FLUO-4 AM staining and ICP-MS. For the FLUO-4 AM assay, 4T1 cells were plated in confocal dishes (2 × 10^5^ cells/dish) and incubated at 37 °C for 24 h. The medium was then substituted with a fresh solution containing 40 mΜ Ca^2+^ and the respective treatments: PBS, RP (1 × 10^9^ CFU/mL), AA-Lip (1.0 mM), or RP@AA-Lip. Samples designated for NIR exposure were first incubated for 3 h and subsequently irradiated with an 808 nm laser (1.0 W/cm^2^, 5 min). Following an additional 6-hour incubation, cells were rinsed with cold PBS, stained with FLUO-4 AM, and visualized by CLSM. In parallel, for ICP-MS quantification, 4T1 cells were exposed to the Ca^2+^ (40 mΜ)-supplemented medium containing PBS, RP (1 × 10^9^ CFU/mL) + NIR, AA-Lip (1.0 mM), or RP@AA-Lip + NIR. The NIR-treated groups underwent the identical laser irradiation protocol after a 3-hour incubation. After a further 6 h, cells were harvested for cell counting and subsequent ICP-MS analysis.

**Cellular ROS, HSP70 and ATP assessment:** The intracellular ROS, HSP70 and ATP levels in 4T1 cells were measured via DCFH-DA fluorescent probe, western blotting and ATP bioluminescent assay kit. After the cells undergo the same treatment as described in “Determination of intracellular Ca^2+^ level”, the supernatant in the wells was discarded and the cells were carefully washed three times using PBS. The cells were subjected to a 20 min staining procedure at 37 °C using DCFH-DA, and fluorescence in the cells was assessed through CLSM and flow cytometry, respectively. For HSP70 detection, protein extracts were prepared from lysed cells. Following separation on a 10% SDS-PAGE gel and subsequent transfer to a PVDF membrane, the extracted proteins were analyzed by immunoblotting. The membrane was first probed with an anti-PMCA4 primary antibody overnight at 4 °C. After three wash cycles, a one-hour incubation with a suitable HRP-linked secondary antibody was performed at room temperature. The target protein bands were finally visualized via ECL luminescence. For ATP detection, the cells were collected, lysed and centrifuged, and the resulting supernatant was analyzed according to the protocol of the ATP bioluminescent assay kit.

**Detection of mitochondrial membrane potential (MMP):** The status of MMP in vitro after indicated treatments was evaluated through JC-1 staining and flow cytometry. 4T1 cells subjected to the same treatment as described in “Determination of intracellular Ca^2+^ level”, the supernatant in the wells was discarded and the cells were carefully washed three times using PBS. The cells were subjected to a 20 min staining procedure at 37 °C using JC-1, and fluorescence in the cells was observed through CLSM or collected to analyze by flow cytometry.

**Staining of p-MLC:** Confocal dishes were seeded with 4T1 cells at 1×10^5^ per dish, and the cultures were maintained for 24 hours before being separated into three groups. These groups included a control, an RP@AA-Lip + NIR group (RP: 1 × 10^9^ CFU/mL; AA: 1.0 mM), and a group receiving RP@AA-Lip + NIR + 10 μM BAPTA‑AM. After the respective treatments, the cells were fixed in 4% paraformaldehyde for 15 min. Next, the samples were made permeable by exposure to 0.3% Triton X‑100 for 10 min, followed by a 30‑min blocking step using goat serum. The cells were then incubated overnight at 4 °C in the dark with a monoclonal antibody targeting p‑MLC (supplied by Cell Signaling Technology, USA). Afterwards, a secondary antibody, FITC‑labeled goat anti‑mouse IgG, was applied at 37 °C for one hour. DAPI was used to stain the nuclei, and finally the slides were observed with a CLSM.

**Detection of cell mitochondrial morphology and cell–cell junctions:** Bio-TEM was utilized to evaluate the disruption of cell mitochondrial morphology and intercellular junctions by RP@AA-Lip + NIR. Cells were inoculated into a 100 mm culture dish and incubated for 24 h. Then, the culture medium was replaced with fresh Ca^2+^ (40 mΜ)-contained medium involving PBS or RP@AA-Lip (RP: 2 × 10^8^ CFU/mL, AA: 1.0 mM). The NIR groups were incubated for 3 h and then irradiated with a NIR laser. Following an additional 6 h incubation, the supernatant in the wells was discarded, and the adherent cells were fixed for 5 min using 2.5% glutaraldehyde. After being gently detached with a cell scraper, the samples were centrifuged at 2000 r/min for 2 min. The collected cell pellet was carefully resuspended in fresh 2.5% glutaraldehyde without aspiration and underwent further fixation for 30 min in the dark, followed by storage at 4 °C. Finally, the processed samples were prepared and examined using Bio-TEM.

**Destructing cell–cell junctions in vitro:** Cells were inoculated into a 100 mm culture dish and incubated for 24 h. Then, the culture medium was replaced and the cells were then exposed to the Ca^2+^ (40 mΜ)-supplemented medium containing PBS, RP (2 × 10^9^ CFU/mL) + NIR, AA-Lip (1.0 mM), or RP@AA-Lip + NIR. The NIR-treated groups underwent the identical laser irradiation protocol after a 3-hour incubation. After a further 3 h, a gentle pipetting action was applied to dissociate any loosely attached cells. The resulting cell suspension in the supernatant was collected and subjected to staining with fluorescein isothiocyanate (FITC). The absolute quantification of these detached cells via flow cytometry, and the total cell numbers in corresponding samples were measured using a cell counter.

**Wound healing assay:** To assess the migratory capacity of 4T1 cells following RP@AA-Lip + NIR treatment, a wound healing assay was performed. A confluent monolayer of 4T1 cells cultured in a 24-well plate was scratched uniformly using a sterile 200 μL plastic pipette tip. After rinsing twice with PBS, the cells were treated with RP@AA-Lip + NIR (RP: 1 × 10^9^ CFU/mL, AA: 1.0 mM). Cell migration into the wound area was monitored and documented using CLSM.

**Transcriptomic Analysis:** 4T1 cells were plated in 100 mm culture dishes at a density of 5 × 10^6^ cells per dish and maintained at 37 °C for 24 hours. Following this, the cells were exposed to the Ca^2+^ (40 mΜ)-supplemented medium containing PBS, RP (2 × 10^9^ CFU/mL) + NIR, AA-Lip (1.0 mM), or RP@AA-Lip + NIR. The NIR-treated groups underwent the identical laser irradiation protocol after a 3-hour incubation. After treatment, cells were harvested with scrapers and immediately snap-frozen in liquid nitrogen. High-throughput RNA sequencing was conducted by Biomarker Biotechnology Co., Ltd., and the resulting data were analyzed via an interactive bioinformatics platform. Differentially expressed genes were considered statistically significant based on a fold change > 2 or a fold change < 0.5 and a P-value < 0.05.

**Detection of ICD:** The surface CRT expression and the extracellular amount of HMGB1 were assessed under in vitro conditions. 4T1 cells were plated in glass-bottom culture dishes at a density of 3 × 10^5^ cells per dish and allowed to adhere for 24 hours. The medium was then substituted with a 40 mΜ Ca^2+^-supplemented solution containing PBS, RP (2 × 10^9^ CFU/mL), AA-Lip (1.0 mM), or RP@AA-Lip. Samples designated for NIR exposure were first incubated for 3 h and subsequently irradiated with an 808 nm laser (1.0 W/cm^2^) for 3 min. To visualize CRT exposure and HMGB1 release, immunofluorescence staining was performed: after fixation with paraformaldehyde, samples were incubated with primary antibodies targeting CRT or HMGB1 at 4 °C overnight, followed by staining with Alexa Fluor 488-conjugated goat anti-mouse IgG (H+L) at 37 °C for one hour. Fluorescence imaging was carried out using CLSM. Meanwhile, CRT exposure levels after PBS, RP, or AA-Lip, RP@AA-Lip with/without NIR treatments were detected using flow cytometry. Concurrently, culture supernatants were harvested, and HMGB1 levels in the medium were measured using a commercial ELISA. In addition, CRT exposure and HMGB1 release from 4T1 cells receiving RP@AA-Lip + NIR + 10 μM BAPTA‑AM treatment also evaluated by immunofluorescence staining.

**DC maturation in vitro:** Bone-marrow-derived dendritic cells (BMDCs) were extracted from C57BL/6 mice and cultured as in the previous studies. In a transwell co-culture setup, DCs were plated in the lower chamber, while 4T1 tumor cells subjected to various pretreatment conditions similar to “Determination of intracellular Ca^2+^ level” were introduced into the upper compartment and co-cultured for 24 hours. DCs from each group were then collected and stained for flow cytometric analysis using fluorescently labeled antibodies against CD80, CD86, and CD11c. In parallel, culture supernatants were harvested, and the levels of the pro-inflammatory cytokines IL-6, IL-12p70, and TNF-α released by DCs were quantified with corresponding ELISA kits.

**Animal model:** The 4T1 murine breast cancer model was established via a single subcutaneous injection of 100 μL cell suspension (1×10^7^ cells/mL) into the right flank. Following model induction, all animals were randomly assigned to the designated study cohorts. All experimental methodologies adhered to the ethical guidelines and were approved by the Henan Laboratory Animal Center.

**In vivo distribution investigation:** When the tumor volumes close to about 200 mm^3^, the IR780 and RP-IR780@AA-Lip (IR780: 2.5 mg/kg) were injected into the mice intravenously. At predetermined time points, the mice were anesthetized and imaged by the IVIS imaging system. After 48 h of intravenous injection with the IR780 and RP@IR780-Lip, the mice were sacrificed to collect tumors and main organs for fluorescence imaging analyses.

**In vivo tumor penetration:** To assess the tumor penetration ability of RP@AA-Lip in vivo, 4T1 tumor-bearing mice were intravenously administered IR780 and RP-IR780@AA-Lip (at an IR780 equivalent dose of 2.5 mg/kg) and maintained for 24 h prior to NIR irradiation (1 W/cm^2^, 5 min). Following an additional 24-hour period, the mice were euthanized, and tumor tissues were collected, flash-frozen, and sectioned. These sections were then subjected to staining with DAPI and an anti-CD31 antibody, and finally visualized by CLSM.

**Antitumor effect in vivo:** The 4T1 tumor-bearing Balb/c mice were randomly divided into eight groups after the tumor volumes were close to 100 mm^3^ (n = 15), and treated with 1) PBS, 2) PBS + NIR, 3) RP, 4) RP + NIR, 5) AA-Lip, 6) AA-Lip + NIR, 7) RP@AA-Lip, 8) RP@AA-Lip + NIR (RP = 2 × 10^9^ CFU/mL, AA = 3 g/kg). For the NIR groups, the mice were irradiated by an 808 nm laser (1.0 W/cm^2^) for 5 min after being intravenously injected 24 h. The tumor volume and body weight of mice were recorded every two days, and the equation (tumor width)^2^ × (tumor length)/2 was utilized to calculate tumor volume.

On day 8, tumors were harvested from three mice per group and subsequently digested with 0.05% collagenase type IV to obtain a single-cell suspension. Intracellular Ca^2+^ levels in the resulting tumor cells were then quantified by ICP-MS.

On day 14, tumors, main organs (heart, liver, spleen, lung, kidney), and blood were collected from the sacrificed mice for pathological and biochemical analysis. Pathology analysis of the organs was conducted using H&E staining assays. The blood biochemistry analyses were performed to assess hepatic and renal functions. The tumors were fixed in 4% of formaldehyde for pathology analysis via ROS, HSP70, CAPN1, E-Cadherin, H&E, TUNEL and Ki67 staining.

**Determination of survival time:** Once the tumor volume attained approximately 100 mm^3^, the 4T1-bearing Balb/c mice were randomly allocated into eight groups (n = 6) and treated with 1) PBS, 2) PBS + NIR, 3) RP, 4) RP + NIR, 5) AA-Lip, 6) AA-Lip + NIR, 7) RP@AA-Lip, 8) RP@AA-Lip + NIR respectively. For the NIR groups, the mice were irradiated by 808 nm laser (1.0 W/cm^2^) for 5 min after being intravenously injected 24 h. After 14 days of treatment, the mice were not euthanized and continued to be housed under the same conditions for a total of 50 days. The status and number of surviving mice during this period were recorded (death was considered when the tumor volume reached 2000 mm^3^).

**Immune response in vivo:** After three treatments, the tumors from all groups were isolated to analyze CD3+CD8+ T cells, CD3+CD4+CD25+FOXP3+ Treg cells by flow cytometry. The TDLNs from all groups were isolated to analyze CD11c+CD86+CD80+ DCs. The spleens from all groups were isolated to analyze CD3+CD8+ T cells. In addition, inflammatory cytokines IFN-𝛾 and TNF-𝛼 in serum from mice after various treatments were measured using ELISA kits following the manufacturer’s instructions. The expression of CRT and secretion of HMGB1within the tumor after PBS, RP, AA-Lip, or RP@AA-Lip with/without NIR treatments were evaluated by immunofluorescence staining.

**Statistical analysis:** All results in this study were recorded as mean ± SD. Statistical analysis in multiple groups was performed by the one-way ANOVA to identify significant differences. Statistical analysis in two groups was performed by an unpaired Student’s t-test. Survival comparisons among experimental groups were performed using the Log‑rank (Mantel‑Cox) test. Differences were considered significant when **P* < 0.05, ***P* < 0.01 and ****P* < 0.001.

Figure S1. Photothermal performance assessment of RP. (A) The photothermal effect of RP with different concentrations; (B) The photothermal effect of RP under different irradiation powers; (C) The photothermal conversion efficiency of RP; (D) The photothermal stability of RP.

Figure S2. UV absorption spectra of RP after different irradiation times.

Figure S3. (A) HPLC standard curve of AA. (B) EE and LC of AA at different feeding amount of AA (n = 3). (C) EE and LC of AA at different feeding amount of cholesterol (n = 3).

Figure S4. Size distribution and Zeta potential of RP@AA-Lip in 10% FBS culture medium for 24 and 48 h (n = 3). (B) The AA leakage rate in 10% FBS culture medium for 48 h (n = 3).

Figure S5. (A) CLSM images of DCFH-DA after PBS, RP, AA-Lip, or RP@AA-Lip with/without NIR treatments. Scale bar: 100 μm. (B) Relative fluorescence intensities of ROS in 4T1 cells after PBS, RP, or AA-Lip, RP@AA-Lip with/without NIR treatments (n = 3).

Figure S6. Fluorescence images of 4T1 cells after PBS, RP, or AA-Lip, RP@AA-Lip with/without NIR treatments for live/dead evaluation. Live (Calcein AM, green) and dead (PI, red). Scale bar: 100 μm.

Figure S7. The results of flow cytometry obtained from 4 T1 cells staining with Annexin V‑FITC/PI after PBS, RP, or AA-Lip, RP@AA-Lip with/without NIR treatments.

Figure S8. CLMS images of 4T1 cells after PBS, RP, or AA-Lip, RP@AA-Lip with/without NIR treatment for Ca^2+^ detection. cell nucleus (Hoechst 33342, blue), Ca^2+^ content (Fluo-4 AM, green). Scale bar: 20 μm.

Figure S9. CLMS images of NIH3T3 cells after PBS, RP, or AA-Lip, RP@AA-Lip with/without NIR treatment for Ca^2+^ detection. cell nucleus (Hoechst 33342, blue), Ca^2+^ content (Fluo-4 AM, green). Scale bar: 20 μm.

Figure S10. CLSM images and flow cytometry analysis of ΔΨm in 4T1 cells after different treatments. Scale bar: 100 μm.

Figure S11. ATP content in 4T1 cells after PBS, RP, AA-Lip, or RP@AA-Lip with/without NIR treatments (n = 3).

Figure S12. The p-MLC fluorescence image of 4T1 cells with different treatments. Scale bar: 20 μm.

Figure S13. Western blot and quantitative analysis of E-Cadherin of various groups after different treatments (n = 3).

Figure S14. The wound healing of 4T1 cells at 0 h, 12 h and 24 h after indicated treatments. Scale bar: 100 μm.

Figure S15. Volcanic diagram of DEGs among different groups (n = 3).

Figure S16. Circle plot of the GO enrichment analysis in 4T1 cells treated with control group and RP@AA-Lip + NIR group (n = 3).

Figure S17. Chord diagram of the GO analysis between RP@AA-Lip+NIR and control groups (A: Biological process; B: Molecular function; C: Cellular component) (n = 3).

Figure S18. (A) Immunofluorescence images of CRT in 4T1 tumor cells after RP@AA-Lip + NIR and RP@AA-Lip + NIR +BAPTA-AM treatment. (C) Immunofluorescence images of HMGB1release in 4T1 tumor cells after RP@AA-Lip + NIR and RP@AA-Lip + NIR +BAPTA-AM treatment.

Figure S19. Peripheral blood plasma C-reactive protein (CRP) and procalcitonin (PCT) levels were detected at 24 h and day 7 between control animals and RP‑receiving animals (n = 3).

Figure S20. (A) CLSM images and the corresponding mean fluorescence intensity (MFI) of Cy3-labeled RP and (B) RP@AA-Lip under normoxia vs hypoxia conditions after various incubation times (n = 3).

Figure S21. Representative H&E-stained tumor tissue evaluating the therapeutic effects of PBS, RP, AA-Lip, or RP@AA-Lip with/without NIR irradiation. Scale bar: 100 μm.

Figure S22. Body weight changes of mice during treatments (n = 5).

Figure S23. H&E staining of major organs in each group of mice (Scale: 200 μm).

Figure S24. The blood levels of white blood cells (WBC), red blood cells (RBC), platelet (PLT), hemoglobin (HGB), and serum biochemical markers of alanine aminotransferase (ALT), aspartate aminotransferase (AST) and blood urea nitrogen (UREA) and creatinine (CREA) of tumor-bearing mice in different groups were analyzed (n = 3).

Figure S25. Flow cytometry gating strategy for the analysis of DCs maturation in vivo.

Figure S26. (A) Flow cytometry gating strategy for the analysis of CD8+ T cells in vivo. (B) Flow cytometry histogram and the corresponding quantitative data of CD3+CD8+ CTLs within spleen after PBS, RP, AA-Lip, or RP@AA-Lip with/without NIR treatments (n = 3).

Figure S27. Flow cytometry gating strategy for the analysis of Tregs in vivo.

Table S1. Blood routine and blood chemistry of the BALB/C mice in experimental group and control group (n = 3).

Table S2. The weight changes of BALB/C mice on day 0 and 30 in experimental group (n = 5).
